# Lactose‐Derived Carbohydrates Induce Sexually Dimorphic Nutritional Programming Effects on Lifespan in *Drosophila melanogaster*

**DOI:** 10.1111/acel.70429

**Published:** 2026-02-26

**Authors:** Peixin Sun, Shiying Shao, Robin W. Creemers, Anna F. Bekebrede, Jing Tang, Steven Driever, Jaap Keijer, Evert M. van Schothorst

**Affiliations:** ^1^ Human and Animal Physiology Wageningen University and Research Wageningen the Netherlands; ^2^ State Key Laboratory of Animal Nutrition and Feeding, Institute of Animal Science Chinese Academy of Agricultural Sciences Beijing China; ^3^ Centre for Crop Systems Analysis Wageningen University and Research Wageningen the Netherlands

**Keywords:** developmental origins of health and disease, dietary carbohydrates, fruit fly, lactose, lipid metabolism, longevity, phospholipids

## Abstract

Early‐life nutrition can exert long‐lasting effects on later‐life health. Given that lactose is extensively consumed during early mammalian development, this raises the intriguing possibility that lactose or its constituent galactose may exert beneficial nutritional programming effects. We tested here whether early‐life (larval period) co‐consumption of galactose and glucose (GALGLU; as in hydrolysed lactose) shapes later‐life (adult) lifespan in *Drosophila melanogaster* . Larval GALGLU versus isocaloric glucose consumption (GLU) significantly extended the developmental time of larvae, increased the pupal volume, decreased pupal oxygen consumption, and reduced the pupal mitochondrial mass. These early‐life effects were translated into sexually dimorphic effects on adult lifespan. Specifically, larval GALGLU consumption extended the lifespan of females when challenged with an obesogenic adult diet, whereas it reduced lifespan in males. To identify molecular correlates of the female‐specific benefit, we profiled transcriptomes and lipidomes. Notably, larval GALGLU induced later‐life transcriptional activation of cuticular hydrocarbon (CHC)‐synthesizing enzymes, including the diene‐producing desaturase *Fad2*, without changes in the monounsaturated fatty acid (MUFA)‐producing desaturase *Desat1*, indicating increased MUFA demand without increased supply. Lipidomic analysis revealed decreased MUFA‐containing and increased polyunsaturated fatty acid (PUFA)‐containing glycerophospholipids. These data suggest that enhanced CHC biosynthesis depletes cellular MUFAs, driving compensatory incorporation of PUFAs into glycerophospholipids. Concluding, early‐life galactose and glucose co‐consumption programs sexually dimorphic lifespan, specifically by counteracting the lifespan‐shortening effects of obesogenic diets in adult females, and redirects adult female lipid metabolism toward a PUFA‐enriched glycerophospholipid profile.

## Graphical abstract

Early‐life co‐consumption of galactose and glucose (hydrolyzed lactose) programs *Drosophila* *melanogaster* lifespan in a sex‐specific manner. Particularly, the co‐consumption of galactose and glucose programs extended lifespan when female flies were challenged by obesogenic diets later in life, and this is concomitant with remodeling of lipid metabolism, based on transcriptomics and lipidomics.

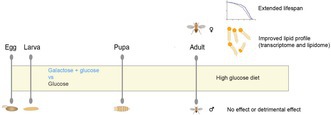

## 1 Introduction

Nutrition is a key determinant of health and lifespan in many animal species (Fontana and Partridge 2015). Notably, early‐life nutrition during the critical developmental windows ranging from the prenatal to the postnatal period in humans exerts long‐lasting effects on adult health (Hoffman et al. 2021), also known as nutritional programming, prompting calls to optimize nutrition during the first 1000 days in humans (Schwarzenberg et al. 2018). Carbohydrate quantity in early life is known to exert long‐lasting effects on later‐life health; for example, early‐life low sugar consumption improves adult metabolic health in humans (Gracner et al. 2024), and early‐life high sugar consumption shortens lifespan in fruit flies (Dobson et al. 2017). However, little is known about whether the type of carbohydrates in early life also shapes adult health.

Lactose is an important carbohydrate in early‐life mammalian postnatal nutrition since it is the major carbohydrate in milk. The digestibility of lactose is limited by the availability of lactase, narrowing its application. This milk sugar is a disaccharide composed of equimolar amounts of the monosaccharides glucose and galactose. Glucose is one of the primary dietary monosaccharides across most life stages, while galactose strikingly is “reserved” for early‐life nutrition. Galactose accounts for almost 50% of the carbohydrate intake during exclusive breastfeeding but becomes substantially reduced in later life after weaning, although adult humans continue to ingest small amounts from dairy and other foods.

Isocaloric substitution of dietary glucose or fructose by galactose improved insulin sensitivity in rats (Stahel et al. 2017). Compared to glucose, equimolar galactose and glucose, mimicking hydrolysed lactose, in the postweaning diet alleviated hepatic inflammation and reduced hepatic triglyceride content in mice (Bouwman, Swarts, et al. 2019). Furthermore, equimolar galactose and glucose, versus glucose in the postweaning diet for 3 weeks, decreased fat mass (Bouwman, Fernández‐Calleja, et al. 2019) and increased circulating insulin‐sensitizing adiponectin levels (Sun et al. 2022) after nine subsequent weeks on an obesogenic diet. These findings highlight the potential beneficial programming effects of galactose and glucose co‐consumption on metabolic health in young adult mice. However, it remains unclear whether co‐consumption of galactose and glucose exerts lifelong effects. Therefore, we studied whether early‐life co‐consumption of galactose and glucose, compared to glucose, exerts effects on lifespan and whether it elicits later‐life molecular alterations.

To address these questions, we turned to the fruit fly *Drosophila melanogaster* (*Drosophila*), a powerful model for nutritional programming studies due to its relatively short lifespan, distinct developmental stages, precision manipulation of the diet, and conserved metabolic pathways. Notably, marula fruit, a natural source of galactose (Leakey 1999), is the ancestral food of wild *Drosophila melanogaster* (Mansourian et al. 2018). Therefore, galactose is physiologically relevant for fruit flies, although *Drosophila* laboratory diets typically do not contain galactose (Bass et al. 2007). Indeed, like mammals, *Drosophila* metabolize galactose via the Leloir pathway (Sakizli et al. 2024). In this process, galactose is converted into glucose‐1‐phosphate, which can be used for glycogen synthesis or is converted to glucose‐6‐phosphate for glycolysis. The *Drosophila* fat body, a functional analogue of mammalian liver and white adipose tissue combined, serves as the primary site for galactose metabolism and energy storage (Sakizli et al. 2024). By providing equimolar amounts of galactose and glucose mimicking lactose from breastmilk after intestinal hydrolysis, we tested whether this dietary exposure during the larval period affects adult lifespan and affects later‐life molecular processes in *Drosophila*.

## 2 Materials and Methods

### 2.1 *Drosophila* Stock and Diet Preparation

The stock flies (W^1118^ strain) were housed in *Drosophila* vials (789008, Kisker Biotech, Steinfurt, Germany) with 20 flies per vial, a mixture of males and females, and were maintained at 25°C ± 0.5°C, with a 12‐h light–dark cycle. Diet preparation is based on protocols previously described (Bass et al. 2007; Skorupa et al. 2008). The composition of the diets is shown in Table 1 and they were used as follows. During the larval period, larvae were fed a standard control diet containing glucose (GLU) or a diet containing galactose and glucose in a 1:1 ratio (GALGLU). During the adult period, the flies were fed a GLU, a high‐glucose (HGLU), a standard sucrose (SUC), or a high‐sucrose (HSUC) diet. Groups were termed according to the diets using the naming convention early diet‐later diet (e.g., GLU‐HGLU means *Drosophila* fed GLU diet during larval period and HGLU diet in adulthood). The stock flies were maintained on the GLU diet.

**TABLE 1:** Dietary composition.^a^

Ingredients	GLU	GALGLU	HGLU	SUC	HSUC
Glucose (M)	0.28	0.14	1.12	0	0
Galactose (M)	0	0.14	0	0	0
Sucrose (M)	0	0	0	0.28	1.12
Yeast % (w/v)	10	10	10	10	10
Agar % (w/v)	1.5	1.5	1.5	1.5	1.5
Nipagin % (w/v)	0.13	0.13	0.13	0.13	0.13
Propionic acid % (v/v)	0.3	0.3	0.3	0.3	0.3

^a^ Glucose (G7021, Merck, Amsterdam, The Netherlands); galactose (G0750, Merck); sucrose (V900116, Merck); yeast (7502231540858, De Zuidmolen, Groesbeek, The Netherlands); agar (76050048, Boom, Meppel, The Netherlands); nipagin (H3647, Merck); propionic acid (P1386, Merck).

### 2.2 Synchronized Egg Collection

For each experiment, synchronized eggs were used to ensure all eggs were from the same parental population, with closely matched hatch time (Linford et al. 2013). Synchronized eggs were randomly seeded at a density of 80–120 eggs per vial containing 6 mL of the specified diet. Detailed procedures are described in the Supporting Information; methods.

For each experimental cohort, vials from different dietary groups were evenly distributed within the tray to minimize potential positional effects. One vial was treated as one biological replicate unless otherwise specified.

### 2.3 Larval Developmental Time and Pupal Size

Synchronized eggs were seeded on GLU or GALGLU diets (12 vials per dietary group), with each vial treated as one biological replicate. Eggs in each vial were counted under a microscope (egg numbers are shown in Figure S1A), and the time to pupation was recorded every 5 h during the daytime (07:30–18:30 h). The ratio of pupae to original eggs was used to generate early‐life developmental curves, which fit a 4‐PL curve, from which the time that 50% of eggs formed pupae was interpolated. The maximum ratio indicated the survival rate during the larval period. The numbers of eclosed flies were recorded to calculate the survival rates from pupae to adults.

Pupae were removed from vials (20 pupae per vial; 9–10 vials per dietary group) within 1 h after pupae formation, placed on graph paper, and photographed under a microscope. Pupal length and width were measured individually using ImageJ software. The pupal volume was calculated based on the equation:

$$\text{Pupal volume}=4/3\pi \left(\frac{L}{2}\right){\left(\frac{D}{2}\right)}^{2}$$

(*L*, length; *D*, diameter) (Delanoue et al. 2010). Each vial was treated as one biological replicate. The pupal volume of 20 collected pupae from the same vial was averaged into a single value.

### 2.4 Pupal Oxygen Consumption

Synchronized eggs were seeded on GLU or GALGLU diets. Pupae formed within 1 h from both GLU and GALGLU diets were used for oxygen consumption measurements. Oxygen consumption was measured using a Differential O_2_ Analyzer system (Qubit Systems, Kingston, Canada) with two 10 mL insect‐specific chambers. One chamber was used as the reference chamber and 25 pupae were placed in the other chamber. The oxygen concentration difference (Delta concentration) between the two chambers was recorded per second. The oxygen consumption for each sample was measured over a 20‐min period to reach a stable delta concentration. The oxygen consumption per pupa was calculated based on the following formula:

$$\text{Oxygen consumption}\;\left(\frac{\mathrm{\mu L}}{\min}\right)=\frac{D\text{elta concentration}\;\left(\mathrm{ppm}\right)\times \mathrm{Air}\;\text{flow}\;\left(\frac{\mathrm{\mu L}}{\min}\right)}{\text{Pupae number}}$$

(Air flow: 40000 μL/min; Pupae number: 25 pupae per measurement). The body weight of the pupae was measured as the pooled mass of the 25 pupae in each chamber using an analytical balance (30355500, Mettler Toledo, Tiel, The Netherlands), and the mean individual weight was estimated by dividing by 25. This procedure was repeated 10 times in the GLU group and 7 times in the GALGLU group with different batches of pupae.

### 2.5 Citrate Synthase Assay in Pupae

Synchronized eggs were seeded on GLU or GALGLU diets (8 vials per dietary group), with each vial treated as one biological replicate. Pupae formed within 1 h from GLU and GALGLU diets (5 pupae per vial) were collected immediately and were snap frozen in liquid nitrogen for subsequent citrate synthase measurements. The citrate synthase content was assayed according to the manufacturer's instructions (CS0720, Merck). The citrate synthase content was used as a proxy for mitochondrial mass. A detailed protocol can be found in the supplemental methods.

### 2.6 Body Weight and Whole‐Body Triacylglycerol Measurement

Synchronized eggs were seeded on GLU or GALGLU diets (12 vials per dietary condition), with each vial treated as one biological replicate. Pupae formed within 1 h from GLU and GALGLU diets were collected (approximately 30–40 pupae per vial). The total body weight per vial was measured, and the mean individual body weight was calculated by dividing the total weight by the number of pupae.

Five pupae per vial were snap frozen in liquid nitrogen for triacylglycerol (TAG) content measurement according to (Tennessen et al. 2014) using the TAG assay kit reagents (10720P, Instruchemie, Delfzijl, The Netherlands). The TAG content of pupae was normalized by body weight. A detailed protocol can be found in the supplemental methods.

### 2.7 Lifespan Assay

Synchronized eggs were seeded on early‐life diets (GLU or GALGLU; 12 vials per dietary group). The day of eclosion was defined as day 0. After eclosion, male and female flies from the same dietary group were transferred together to embryo collection cages for 2 days to ensure sexual maturation, at a density of about 400 flies per cage. Male and female flies were then separated under CO_2_ anesthesia and assigned to the different later‐life diets (GLU or HGLU), resulting in four early–later dietary combinations per sex (GLU‐GLU, GLU‐HGLU, GALGLU‐GLU, and GALGLU‐HGLU). For each condition, 20 flies were placed per vial, with 7 vials per condition (approximately 140 flies). To ensure reproducibility, this experiment was performed twice under the same conditions.

An additional experiment was conducted using the same early‐life diets (GLU and GALGLU) plus an additional early‐life control diet containing sucrose (SUC; 12 vials per dietary group). Adult flies were subsequently assigned to later‐life diets containing sucrose (SUC or HSUC), resulting in six early–later dietary combinations per sex (GLU–SUC, GLU–HSUC, GALGLU–SUC, GALGLU–HSUC, SUC–SUC, and SUC–HSUC). For each condition, 20 flies were placed per vial, with 5–7 vials per condition (approximately 100–140 flies).

The adult diets were replaced three times per week, and the mortality was recorded daily as published (Linford et al. 2013). The researcher was blinded to dietary conditions when recording the mortality.

### 2.8 Transcriptomics and Data Analysis

A separate cohort of flies was used for transcriptomic analysis. Twenty‐eight days after eclosion, adult female flies from GLU‐HGLU and GALGLU‐HGLU groups were anesthetized under CO_2_ and dissected in cold, RNase‐free PBS under a stereomicroscope, and the abdominal carcasses were isolated (the dorsal body wall tissue with removal of internal organs like digestive tract and ovary, etc., retaining primarily metabolic organs including fat body and oenocytes) and snap frozen in liquid nitrogen. Each group contained 12 biological replicates (pools of 10 abdominal carcasses per replicate). More detailed information for RNA sequencing and data pre‐processing is described in the Supporting Information; methods.

After the read alignment and counting, data analyses and statistical testing were performed in R 4.4, using appropriate Bioconductor packages. Differential transcripts of GALGLU‐HGLU versus control GLU‐HGLU groups were identified using the DE2seq package, using Benjamini–Hochberg (BH) correction for multiple testing (Love et al. 2014) to obtain false discovery rate (FDR). Transcripts with a FDR < 0.1 were used for enrichment analysis in Kyoto Encyclopedia of Genes and Genomes (KEGG) databases using clusterProfiler (Wu et al. 2021). Pathways were considered significantly enriched with a FDR < 0.05. Heatmaps were generated using the ComplexHeatmap packages (Gu et al. 2016), with z‐scores of log2 transformed raw counts. RNA‐Sequencing data have been deposited in Gene Expression Omnibus (GEO), GSE297977.

### 2.9 Real‐Time Quantitative Polymerase Chain Reaction

RNA was used for cDNA synthesis and subsequent real‐time quantitative polymerase chain reaction (RT‐qPCR) as described (Sun et al. 2022), using *ATP synthase*, *subunit C* (*ATPsynC*) and *Cyclophilin 1* (*Cyp1*) as reference genes. Primer sequences and annealing temperatures can be found in Table S3. Data are expressed as a fold change relative to the mean of GLU‐HGLU.

### 2.10 Lipidomics and Data Analysis

An independent cohort of flies was used for lipidomic profiling. Twenty‐eight days after eclosion, adult female flies from GALGLU‐HGLU and GLU‐HGLU groups were anesthetized under CO_2_ and snap frozen in liquid nitrogen, and untargeted lipidomics was conducted for whole‐body homogenates. Each group had 11 biological replicates (pools of 20 flies per replicate). High‐performance Liquid Chromatography–Tandem Mass Spectrometry was used to separate and detect lipids. Detailed procedures are described in the Supporting Information; methods.

Significance is determined when the variable important for the projection (VIP) score obtained from the orthogonal partial least squares‐discriminant analysis model (OPLS‐DA) ≥ 1 and Student's *t*‐test *p* value < 0.05. Heatmaps were generated using the ComplexHeatmap packages (Gu et al. 2016), with *z*‐scores of log2‐transformed peak intensity. The lipid classes and abbreviations are summarized in Table S4. The glycerophospholipid species are named as lipid subclass (chain length: double bonds), for example, PE(18:1/18:3) is a phosphatidylethanolamine (PE) with one monounsaturated fatty acid (MUFA) containing 18 carbons, and one polyunsaturated fatty acid (PUFA) containing 18 carbons. Species lacking specific fatty acid saturation information (e.g., PE(31:2) could contain two MUFAs or one PUFA plus one saturated fatty acid) were excluded for lipid saturation analyses.

### 2.11 Statistical Analysis

The developmental curve was analyzed by two‐way ANOVA. Student's *t*‐test was used for 50% developmental time, survival rate, pupal volume, absolute oxygen consumption, and citrate synthase content when the data passed normality or lognormality test; otherwise, a Mann–Whitney test was used. Adult body weight and whole‐body TAG content were analyzed with a Student's *t*‐test (as datasets from each timeslot are independent) within each timeslot and each sex when the data passed normality or lognormality test, or otherwise, a Mann–Whitney test was used. The survival curve was analyzed using the Kaplan–Meier method. The hazard ratio was based on the log‐rank test of the survival curve. The median survival is defined as the age at which 50% of the population remains alive. The maximum survival refers to the mean lifespan of the longest‐lived 10% of individuals. The average lifespan represents the mean lifespan across the entire population. Data were analyzed in GraphPad Prism, Version 10.5.0 (GraphPad Software Inc., San Diego, USA), and shown as mean ± SD. *p < 0.05, **p < 0.01, ***p < 0.001, ****p < 0.0001.

## 3 Results

### 3.1 GALGLU Diet Affected Developmental Time and Energy Metabolism in Early‐Life

**FIGURE 1:**
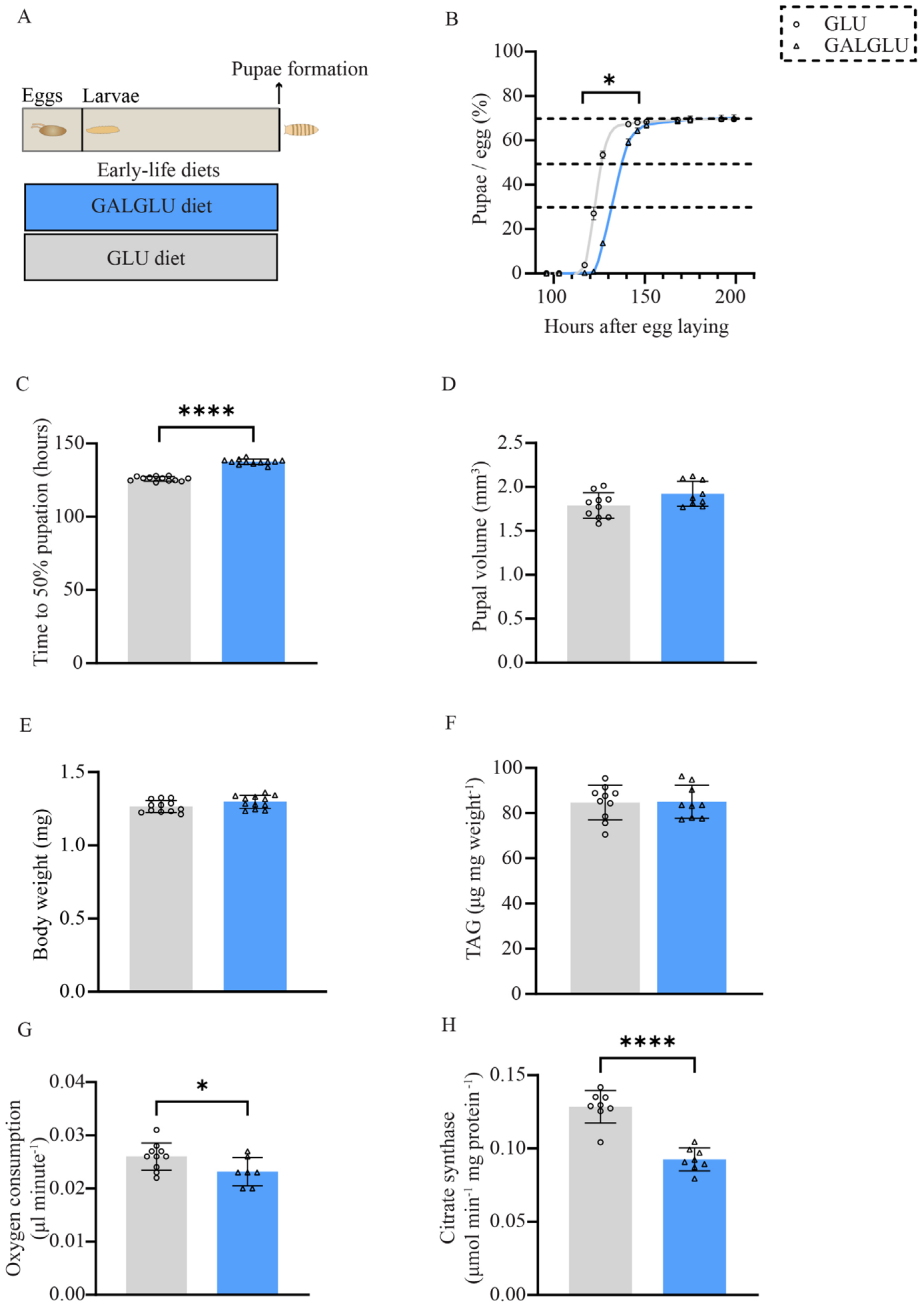
Galactose and glucose co‐consumption regulated early‐life development and metabolism in *Drosophila*. (A) Synchronized eggs were seeded into either 0.14 M galactose + 0.14 M glucose (GALGLU) or 0.28 M glucose (GLU) diets and pupation time was recorded. Upon pupae formation, the volume, body weight, whole body triacylglycerol, oxygen consumption, and citrate synthase content were measured. Upon eclosion, male and female flies were separated, body weight and whole body triacylglycerol were measured. (B) Pupae formation time (*n* = 12 samples in GLU, *n* = 12 samples in GALGLU, with 80–120 eggs per sample). (C) Developmental time from egg laying till 50% of eggs formed pupae, values represent 50% pupation time per sample (*n* = 12 samples in GLU, *n* = 12 samples in GALGLU, with 80–120 eggs per sample). (D) Pupal volume after pupae formation, values represent the average volume per pupa, determined by measuring volumes of 20 pupae and dividing the total by 20 (*n* = 10 samples in GLU, *n* = 9 samples in GALGLU, with 20 pupae per sample). (E) Whole‐body weight of pupae (*n* = 12 samples per group, with 30–40 pupae per sample); values represent the average weight per individual, determined by measuring the total body weight and dividing the total by the number of pupae. (F) Whole‐body triacylglycerol (TAG) of pupae (*n* = 10 samples in GLU, *n* = 9 samples in GALGLU, with 5 pupae per sample), the TAG content was normalized by total weight. (G) Oxygen consumption rate of pupae, values represent the average oxygen consumption per pupa per minute, determined by measuring total consumption rate of 25 pupae and dividing the total by 25 (*n* = 10 samples in GLU, *n* = 7 samples in GALGLU, with 25 pupae per sample). (H) Mitochondrial citrate synthase content normalized to protein content in pupae (*n* = 8 samples per group, with 5 pupae per sample). Statistical analysis was performed by two‐way ANOVA for panel B and by Student's *t*‐test for other panels (*p < 0.05; ****p < 0.0001).

Feeding the GALGLU diet versus GLU diet during the *Drosophila* larval periods, schematically shown in Figure 1A, resulted in a significantly longer time in pupation (Figure 1B,C). However, the mean pupal volume (Figure 1D), weight (Figure 1E), and whole‐body TAG content (Figure 1F) were not significantly different. Strikingly, the oxygen consumption reflecting energy expenditure (Figure 1G) and whole body mitochondrial mass (Figure 1H) were significantly decreased in GALGLU‐fed pupae.

At eclosion, the survival rates from pupae to adults were not significantly different between the two groups (Figure S1B). The body weight of adult males and females was significantly higher in the GALGLU‐fed groups (Figure S1C), while the whole‐body TAG content was significantly higher only in male flies (Figure S1D).

### 3.2 Early‐Life GALGLU Diet Consumption Showed Sexually Dimorphic Effects on Adult Lifespan

**FIGURE 2:**
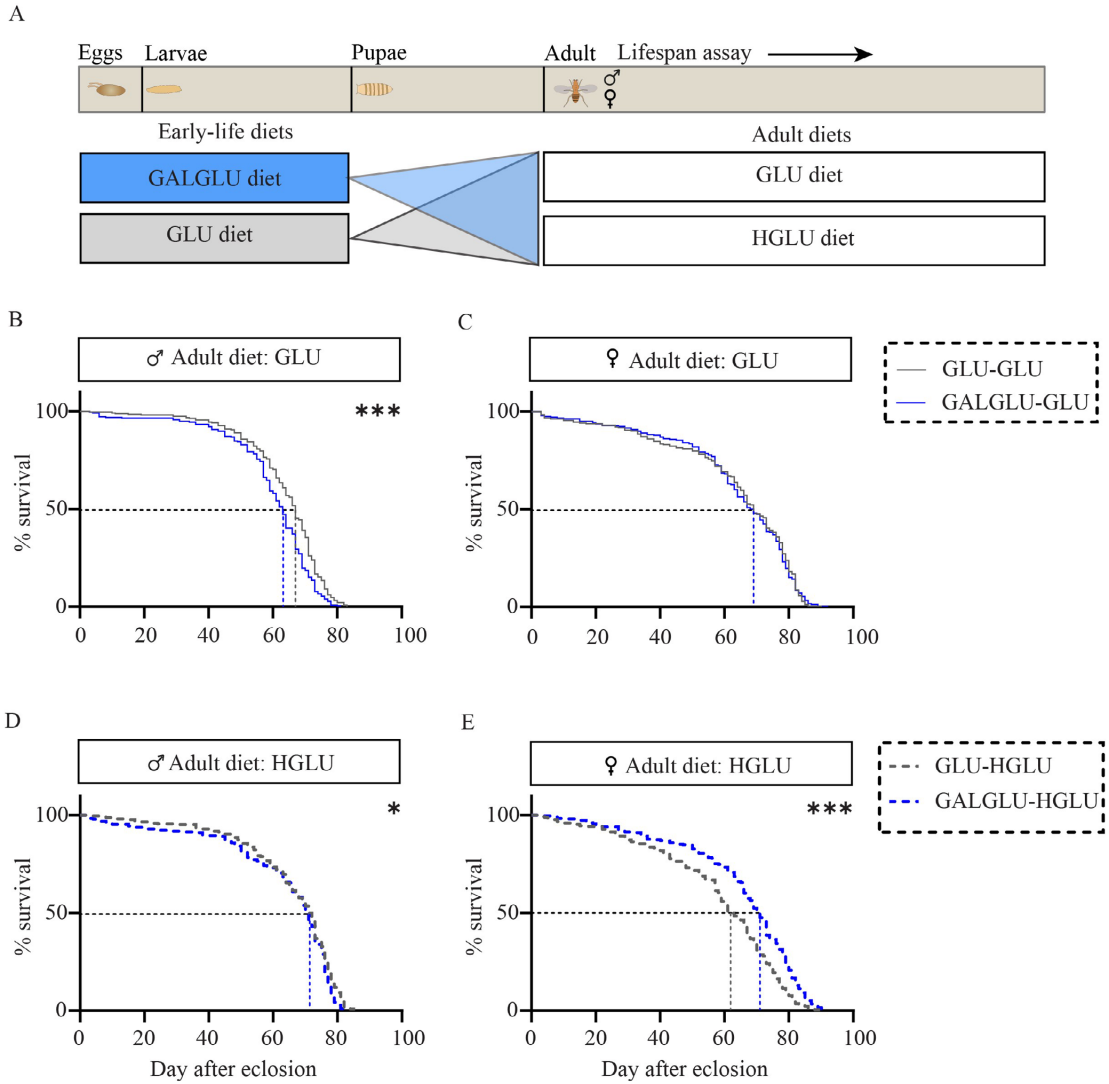
Galactose and glucose co‐consumption in early‐life regulated adult lifespan in *Drosophila*. (A) Scheme for the experiments. Upon feeding either 0.14 M galactose + 0.14 M glucose (GALGLU) or 0.28 M glucose (GLU) diets in early‐life, male and female flies were separately fed GLU diet or 1.12 M glucose (HGLU) diet. The experiments were repeated twice, combined data were shown here. (B) Lifespan curves of male flies (*n* = 276 flies in GLU‐GLU, *n* = 258 flies in GALGLU‐GLU) and (C) female flies (*n* = 243 flies in GLU‐GLU, *n* = 238 flies in GALGLU‐GLU) when maintained on GLU diet. (D) Lifespan curves of male flies (*n* = 271 flies in GLU‐HGLU, *n* = 260 flies in GALGLU‐HGLU) and (E) female flies (*n* = 283 flies in GLU‐HGLU, *n* = 319 flies in GALGLU‐HGLU) when maintained on HGLU diet. Statistical analysis was performed by the log‐rank test (*p < 0.05; ***p < 0.001). See Table S1 for exact statistics. See also Figure S2.

To investigate whether the early‐life (larval) GALGLU diet (vs. GLU diet) had long‐term effects on adult lifespan under either standard (GLU) or obesogenic (HGLU) later‐life dietary conditions, we continued the study, as schematically shown in Figure 2A.

In males, reduced survival in GALGLU‐GLU versus GLU‐GLU fed adults was seen (Figure 2B, Table S1), with a hazard ratio (HR) of 1.43. This detrimental effect persisted (log‐rank test *p* = 0.018) but was attenuated under an obesogenic HGLU dietary condition in adulthood (HR = 1.26; Figure 2D, Table S1).

Females displayed a contrasting response, although the survival curves between GALGLU‐GLU versus GLU‐GLU groups were not significantly different (Figure 2C, Table S1). On an adult HGLU diet, the GALGLU‐HGLU showed an extended median survival by 14.5%, and an increased survival rate compared to the GLU‐HGLU group (log‐rank test *p* < 0.0001; HR = 0.63; Figure 2E, Table S1).

The data shown in Figure 2 are the mean of two independent studies with consistent outcomes (Figure S2), cumulatively showing that early‐life GALGLU diet consumption resulted in sex‐specific effects on adult lifespan. Of note, body weight did not differ significantly between GALGLU‐HGLU and GLU‐HGLU at 7, 14, 21, or 28 days after eclosion in either males or females (Figure S3).

**FIGURE 3:**
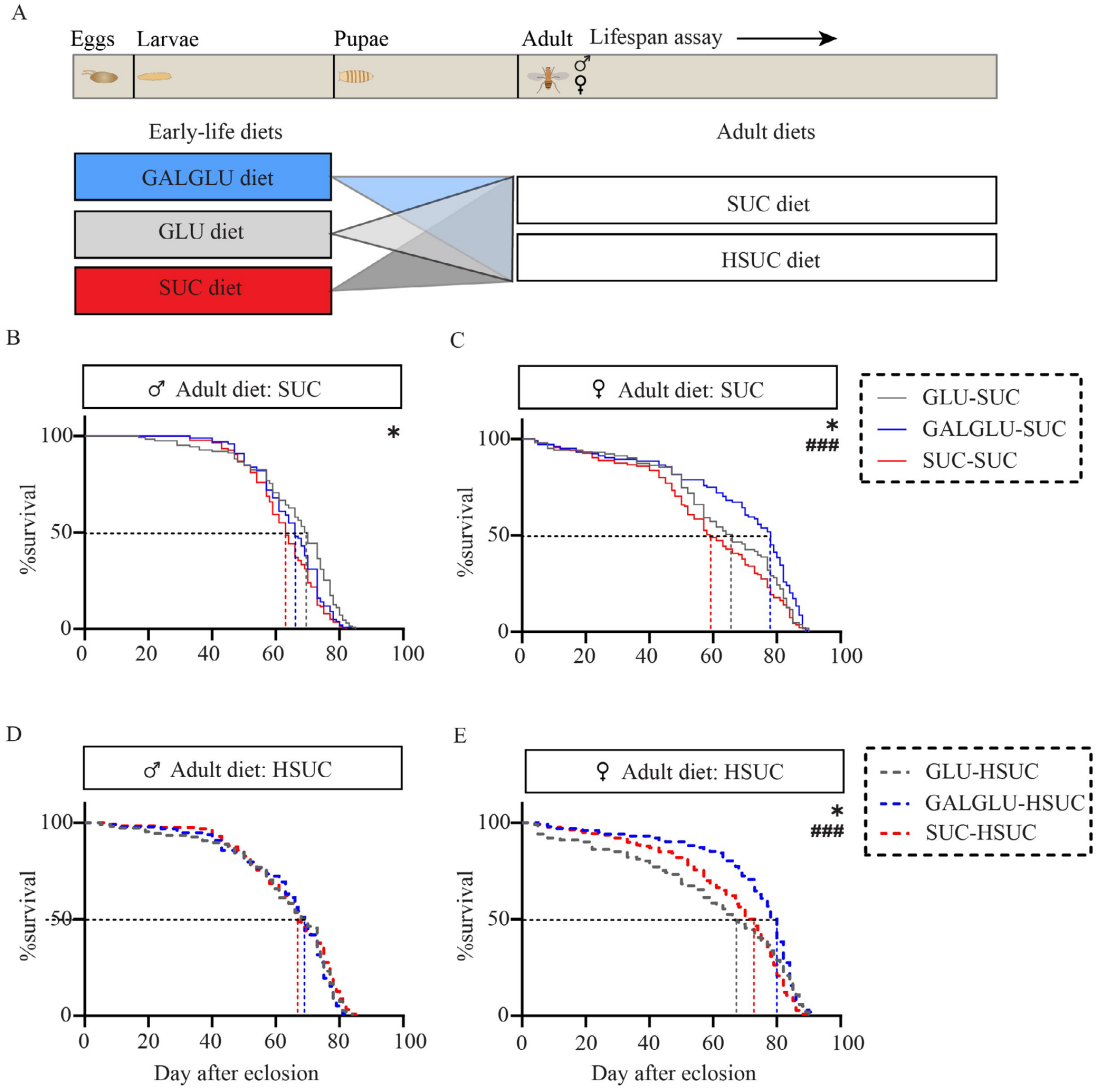
Robust effects of early‐life galactose and glucose co‐consumption on adult lifespan under different dietary contexts in *Drosophila*. (A) Scheme for the experiments. Upon feeding either 0.14 M galactose +0.14 M glucose (GALGLU) or 0.28 M glucose (GLU) or 0.28 M sucrose (SUC) diets in early life, male and female flies were separately fed SUC diet or 1.12 M SUC (HSUC) diet. (B) Lifespan curves of male flies (*n* = 126 flies in GLU‐SUC, *n* = 100 flies in GALGLU‐SUC, *n* = 138 flies in SUC‐SUC) and (C) female flies (*n* = 103 flies in GLU‐SUC, *n* = 104 flies in GALGLU‐SUC, *n* = 135 flies in SUC‐SUC) when maintained on GLU diet. (D) Lifespan curves of male flies (*n* = 108 flies in GLU‐SUC, *n* = 98 flies in GALGLU‐SUC, *n* = 127 flies in SUC‐SUC) and (E) female flies (*n* = 101 flies in GLU‐HSUC, *n* = 102 flies in GALGLU‐HSUC, *n* = 140 flies in SUC‐HSUC) when maintained on HGLU diet. Statistical analysis was performed by the log‐rank test (*p < 0.05 against controls on GLU early‐life diets; ### *p* < 0.0001 against controls on SUC early‐life diets). See Table S2 for exact statistics.

To further investigate the robustness of the observed long‐term effects of early‐life GALGLU diet on adult lifespan, adult flies were fed either a SUC or a HSUC diet, as outlined in Figure 3A. Testing sucrose‐based diets allows comparison with previous studies, as sucrose is another widely used carbohydrate source in *Drosophila* studies (Bass et al. 2007; Skorupa et al. 2008).

In males, the survival‐curve‐based HR was also 34% higher in GALGLU‐SUC than GLU‐SUC (log‐rank test *p* = 0.018; Figure 3B, Table S2), which is comparable to the increased HR seen in GALGLU‐GLU versus GLU‐GLU. An additional group fed the SUC diet also in early‐life showed a survival curve similar to that of the GLU‐SUC groups. This detrimental effect on survival by GALGLU feeding in males was abolished under an obesogenic HSUC challenge in later‐life, without differences among groups (Figure 3D, Table S2).

In females, an increased survival in GALGLU‐GLU versus SUC‐SUC or GLU‐SUC fed adults was seen (Figure 3B, Table S2), with an extended median survival (32% compared to SUC‐SUC; 18% compared to GLU‐SUC) and a reduced mortality risk (38% compared to SUC‐SUC; 27% compared to GLU‐SUC). Strikingly, this protective effect persisted under HSUC challenge in later‐life (all log‐rank test *p* < 0.05); the GALGLU‐HSUC showed an extended median survival of 11%–18% and a reduced mortality risk of 24%–35% compared to SUC‐HSUC and GLU‐HSUC (Figure 3E, Table S2).

Together, these new findings demonstrate that the early‐life (larval) GALGLU dietary intervention exerted sexually dimorphic effects on adult lifespan. While this programming was detrimental in males, it protects females specifically from the life‐shortening effects of the obesogenic adult diet.

### 3.3 Early‐Life GALGLU Diet Consumption Transcriptionally Regulated Very‐Long‐Chain Fatty Acid and Cuticular Hydrocarbon Metabolism in Later‐Life

**FIGURE 4:**
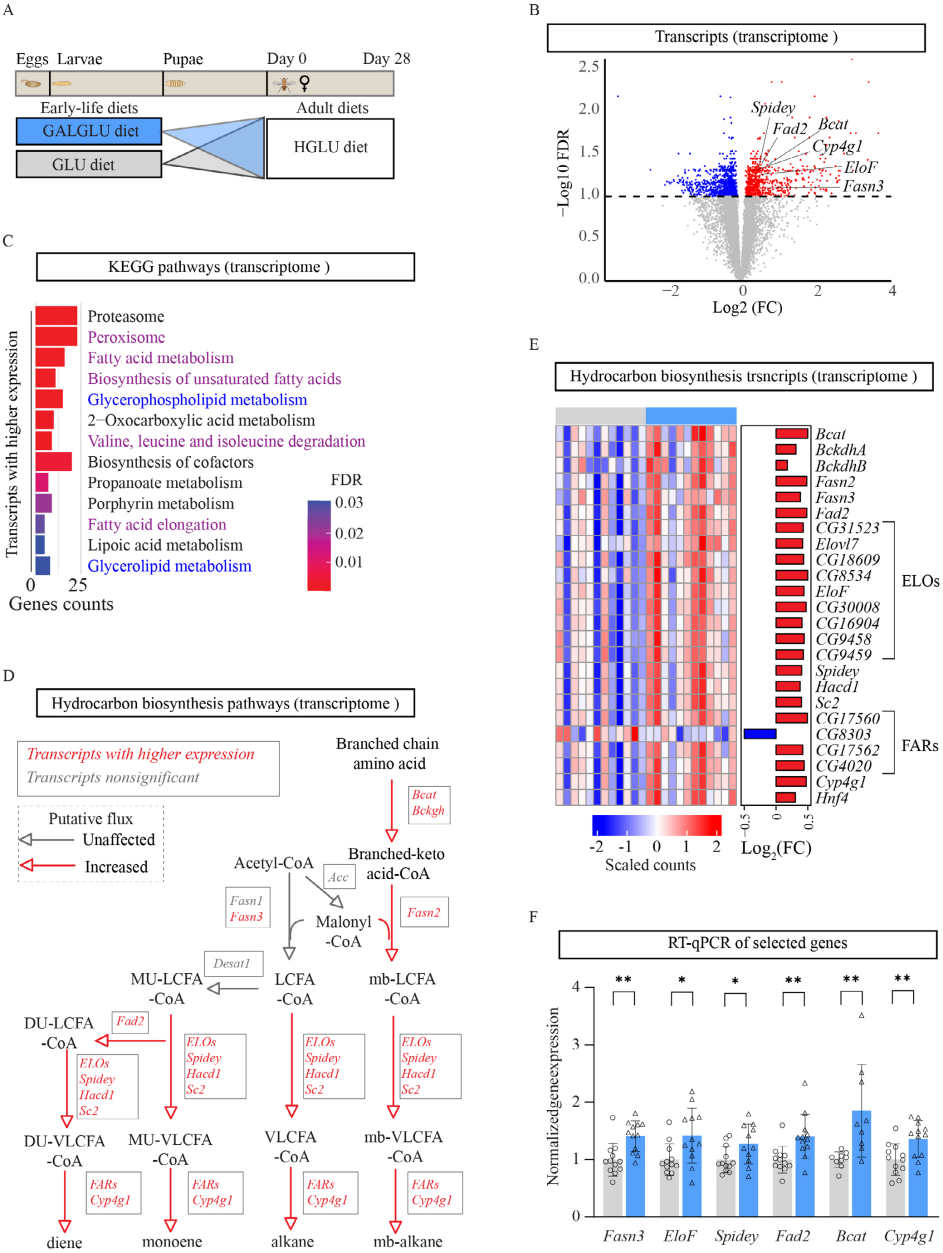
Galactose and glucose co‐consumption in early‐life regulated later‐life lipid metabolism and cuticular hydrocarbon biosynthesis at transcriptional level in *Drosophila*. (A) Scheme for the experiments (also applies to Figures 5 and 6). *Drosophila* were fed 0.28 M glucose diet in early‐life and 1.12 M glucose in later‐life (GLU‐HGLU group), or 0.14 M galactose +0.14 M glucose diet in early‐life and 1.12 M glucose in later‐life (GALGLU‐HGLU group). After eclosion, 28‐day‐old female flies were dissected and RNA‐sequencing was performed on the abdominal carcasses, primarily composed of the fat body and oenocytes (*n* = 12 samples per group, each sample was composed by abdominal carcasses of 10 flies). Whole‐body lipidomics was performed on 28‐day‐old female flies post‐eclosion (*n* = 11 biological replicates per group, 20 flies pooled per sample). (B) Volcano plot, the horizontal dashed line indicates the threshold for significance (FDR < 0.1), red indicates transcripts with higher expression while blue indicates transcripts with lower expression in the GALGLU‐HGLU group, gray indicates non‐regulated transcripts between GALGLU‐HGLU and GLU‐HGLU. (C) Enrichment analysis in Kyoto Encyclopedia of Genes and Genomes (KEGG) database, 13 pathways were enriched by the transcripts with significantly higher expression; pathways highlighted in purple and blue were fatty acid and lipid metabolism related. (D) Simplified cuticular hydrocarbon biosynthesis pathway, adapted from (Pardy et al. 2019) and (Holze et al. 2021). Transcripts are represented as rectangles, red: Transcripts with higher expression; gray: unchanged transcripts. The red arrow suggests a putative increased flux, the gray arrow suggests a putative unchanged flux. LCFA‐CoA, long‐chain fatty acyl‐CoA; VLCFA‐CoA, very‐long‐chain fatty acyl‐CoA; MU, monounsaturated; DU, di‐unsaturated; mb, methyl‐branched; *FARs*, fatty acyl‐CoA reductases‐encoding transcripts; *ELOs*, elongases encoding transcripts. *FARs* and *ELOs* are encoded by multiple transcripts. *Bcat*, *Branched‐chain amino acid aminotransferase*; *Bckdha/b*, *Branched‐chain α‐keto acid dehydrogenase E1 α/β subunit*; *Fasns*, *Fatty acid synthases*; *Fad2*, *desaturase F*; *Elovl7*, *ELOVL family very‐long‐chain fatty acid elongase 7*; *EloF*, *Elongase F*; *CG31523*, *CG18609*, *CG8534*, *CG30008*, *CG16904*, *CG9458*, and *CG9459* are annotated as elongases encoding transcripts in Flybase; *Spidey: 3‐ketoacyl‐CoA reductase*; *Hacd1*, *3‐hydroxyacyl‐CoA dehydratases*; *Sc2*, *trans‐enoyl‐CoA reductase*; *CG17560*, *CG8303*, *CG17562*, *CG4020* are annotated as fatty acyl‐CoA reductases‐encoding transcripts in Flybase; *Cyp4g1*, *cytochrome P450 decarboxylase*; *Hnf4: Hepatocyte nuclear factor 4*; *Acc: Acetyl‐CoA carboxylase*; *Desat1: desaturase 1*; (E) Heatmap created by the scaled counts of key transcripts involved in the described cuticular hydrocarbon biosynthesis pathway, with the log2 (fold change) of GALGLU‐HGLU/GLU‐HGLU. Transcripts were ordered based on their significance. (F) mRNA expression of selected genes involved in the long‐chain fatty acid and hydrocarbon biosynthesis pathways using real‐time quantitative polymerase chain reaction; gene expression was normalized with the expression of *ATPsynC* and *Cyp1*. Statistical analysis in panel F was performed by Student's *t*‐test (*p < 0.05; **p < 0.01).

Given the observed female‐specific beneficial effects on lifespan, we next investigated associated later‐life molecular alterations. We focused on the metabolic organs of the abdominal carcass, primarily composed of the fat body and oenocytes, in 28‐day‐old GALGLU‐HGLU versus GLU‐HGLU female flies (Figure 4A).

Transcriptome analysis revealed 1726 differentially expressed transcripts (FDR < 0.1; Figure 4B), which were used for pathway enrichment analysis in KEGG databases. Transcripts with a significantly lower expression were mainly enriched in genetic information processing pathways (e.g., nucleotide excision repair, DNA replication; Figure S4A). In contrast, transcripts with a significantly higher expression were enriched in 13 pathways, including five fatty acid metabolism‐related pathways (highlighted in purple in Figure 4C) and two glycerophospholipid metabolism‐related pathways (highlighted in blue in Figure 4C). Within the fatty acid metabolism‐related pathways, we observed higher expression of transcripts in peroxisomal fatty acid alpha and beta oxidation (Figure S4B,C), suggesting enhanced branched‐chain fatty acid and very‐long‐chain fatty acid (VLCFA) catabolic processes. Meanwhile, we also found higher expression of transcripts involved in branched‐chain fatty acid anabolic processes (such as *fatty acid synthase 2* [*Fasn2*] and *branched‐chain amino acid aminotransferase* [*Bcat*]) and VLCFA anabolic processes (such as *elongase F* [*EloF*], *3‐ketoacyl‐CoA reductase* [*Spidey*], *3‐hydroxyacyl‐CoA dehydratase 1* [*Hacd1*], and *trans‐enoyl‐CoA reductase* [*Sc2*]) which implicate enhanced cuticular hydrocarbon (CHC) biosynthesis. In line with this, the conserved transcriptional factor *hepatocyte nuclear factor 4* (*Hnf4*), which drives the VLCFA and CHC biosynthesis (Storelli et al. 2019), also showed a higher expression.

Therefore, we mapped the transcriptional data on the previously established (Holze et al. 2021; Pardy et al. 2019) CHC biosynthesis pathway (Figure 4D) and plotted the scaled counts of all significant transcripts in this pathway (Figure 4E). Interestingly, the expression of *Acc* (encodes Acetyl‐CoA carboxylase, the rate‐limiting enzyme for *de novo* fatty acid synthesis) and *Desat1* (encodes desaturase 1, the sole desaturase that introduces the first double bond into acyl‐CoAs in cosmopolitan *Drosophila melanogaster* (Dallerac et al. 2000; Murakami et al. 2017)) was unchanged, potentially suggesting that the *de novo* and monounsaturated fatty acyl‐CoA synthesis remained similar between groups. However, the downstream CHC biosynthesis was likely enhanced, since the rate‐limiting enzyme encoding transcript *Cytochrome P450 4g1* (*Cyp4g1*) showed higher expression. Notably, *Fad2*, the sole desaturase introducing the second double bond in elongated MUFAs (Chertemps et al. 2006; Dembeck et al. 2015), showed higher expression, suggesting increased flux toward diene CHC production. This may systematically reduce the cellular pool of monounsaturated fatty acids available for other processes (e.g., membrane lipid synthesis). The expression of several key transcripts, including *Fasn3*, *EloF*, *Spidey*, *Fad2*, *Bcat*, and *Cyp4g1*, was validated by RT‐qPCR (Figure 4F).

### 3.4 Early‐Life GALGLU Diet Consumption Regulated Glycerophospholipid Profiles in Later‐Life

**FIGURE 5:**
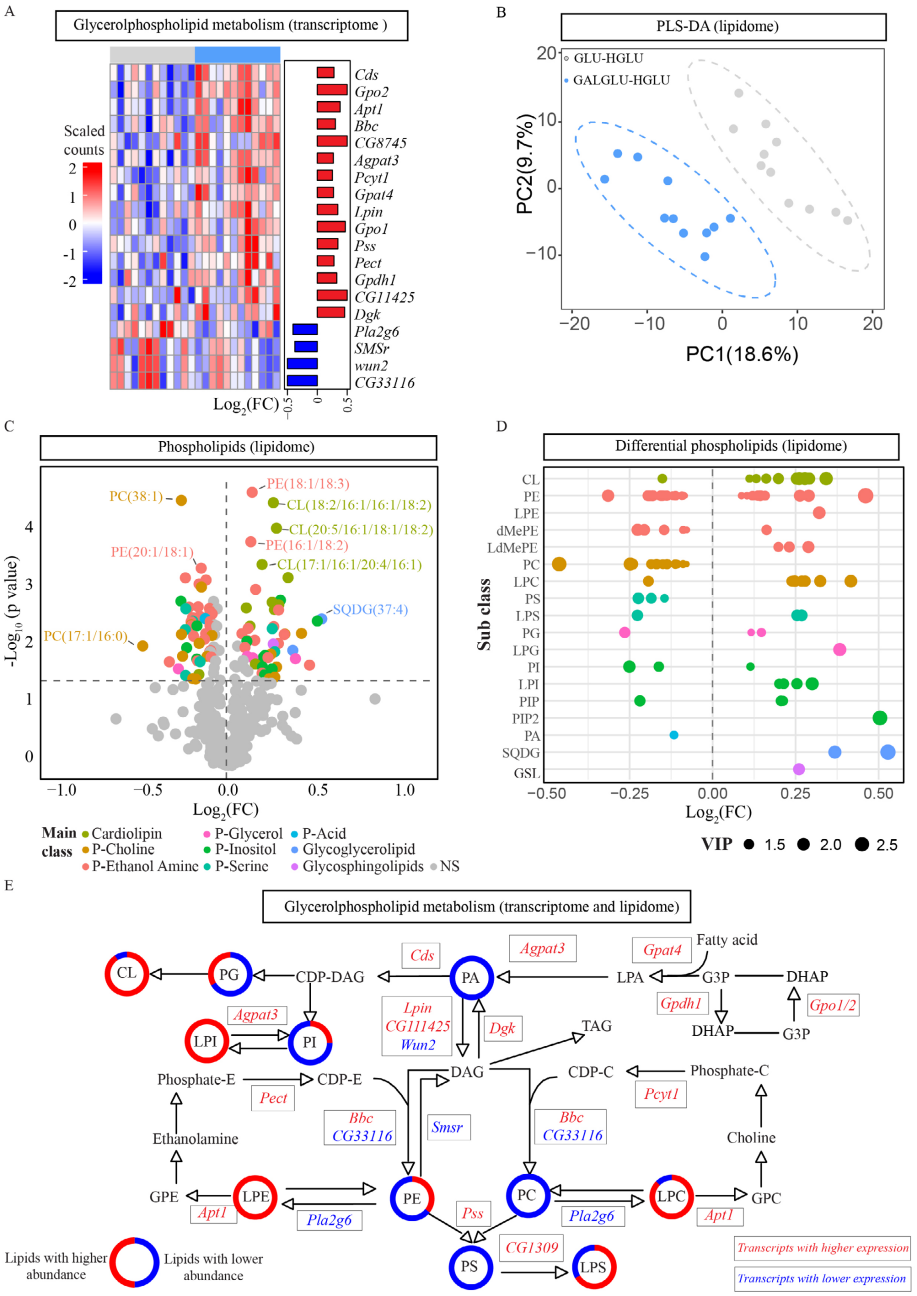
Early‐life co‐consumption of galactose and glucose (GALGLU), versus glucose alone (GLU), shaped later‐life whole‐body glycerophospholipid profile in female *Drosophila* maintained on a high‐glucose adult (HGLU) diet. Lipidomic data derive from the same experimental design outlined in Figure 4A (GALGLU‐HGLU versus GLU‐HGLU). (A) Heatmap created by the scaled counts of key transcripts involved in the glycerophospholipid metabolism pathway (from Kyoto Encyclopedia of Genes and Genomes (KEGG) databases) from the same RNA‐sequencing samples described in Figure 4A, with the log2 (fold change) of GALGLU‐HGLU/GLU‐HGLU. *Cds: CDP‐diacylglycerol synthase*; *Gpo2*, *glycerophosphate oxidase 2*; *Apt1*, *Acyl‐protein thioesterase 1*; *Bbc*, *bb in a boxcar*; *Pla2g6*, *calcium‐independent phospholipase A2* VIA; *Agpat3: 1‐Acylglycerol‐3‐phosphate O‐acyltransferase 3*; *Pcyt1*, *phosphocholine cytidylyltransferase 1*; *Gpat4: Glycerol‐3‐phosphate acyltransferase 4*; *Lpin*, *lipin*; *Gpo1*, *glycerophosphate oxidase 1*; *Smsr*, *sphingomyelin synthase related*; *Pss*, *phosphatidylserine synthase*; *Wun2*, *wunen‐2*; *Pect*, *phosphoethanolamine cytidylyltransferase*; *Gpdh1*, *Glycerol‐3‐phosphate dehydrogenase 1*; *Dgk*, *diacyl glycerol kinase*. (B) PLS‐DA plot, multivariate analysis of whole‐body lipidomic profiles, principle 1 explains 18.6% of the total variation and principle 2 explains 9.7% of the total variation. (C) Volcano plot, differential glycerophospholipid species (colored by main lipid class) between groups. Colored dots: significant species (*p* < 0.05, VIP > 1). Gray dots: non‐significant species. (D) Bubble diagram, subclass‐specific visualization of significant lipid species. Bubble size represents VIP score. The full list of main classes and sub classes can be found in Table S4. (E) Combined transcriptomics and lipidomics data into a simplified glycerophospholipid metabolism pathway from KEGG. Transcripts are represented as rectangles, red: transcripts with higher expression; blue: Transcripts with lower expression. Lipids are represented as donut charts, reflecting the ratio of significant glycerophospholipid species within subclasses (red sectors: higher abundance; blue sectors: Lower abundance). Integration of RNA sequencing data (abdominal carcasses) and lipidomics data (whole‐body homogenates). G3P, glyceraldehyde‐3‐phosphate; LPA, lysophosphatidic acid; PA, Phosphatidic acid; DAG, diacylglycerol; TAG, triacylglycerol; PG, phosphatidylglycerol; PI, phosphatidylinositol; CL, cardiolipin; PC, phosphatidylcholine; PE, phosphatidylethanolamine; PS, phosphatidylserine; LPE, lyso‐phosphatidylethanolamine; LPS, lyso‐phosphatidylserine; LPC, lyso‐ phosphatidylcholine; LPI, lyso‐ phosphatidylinositol; Phosphate‐E, Phosphate‐ethanolamine; Phosphate‐C, Phosphate‐choline; GPC, glycerol‐phosphocholine; GPE, glycerol‐phosphoethanolamine.

We then investigated all significant transcripts in the glycerophospholipid metabolism pathway (KEGG; highlighted in blue in Figure 4C). The significant transcripts suggested an increased fatty acid anabolic flux toward glycerophospholipid metabolism (Figure 5A). Marked by higher expressed key transcripts, such as *CDP‐diacylglycerol synthase* (*Cds*), and *Lpin*, which suggest increased anabolic flux from fatty acid toward diacylglycerol (a precursor for phosphatidylcholine (PC), PE and phosphatidylserine (PS) synthesis) and CDP‐diacylglycerol synthesis (a precursor for phosphatidylglycerol (PG), phosphatidylinositol (PI) and cardiolipin (CL) synthesis). Moreover, the expression of transcripts encoding rate‐limiting enzymes for PC, PE, and PS was also higher, including *bb in a boxcar* (*Bbc*), *phosphocholine cytidylyltransferase 1* (*Pcyt1*), *phosphoethanolamine cytidylyltransferase* (*Pect*), and *phosphatidylserine synthase* (*Pss*).

In light of the transcriptional regulation of the fatty acid and glycerophospholipid metabolism, we next determined the later‐life glycerophospholipid profiles. An untargeted lipidomic analysis was performed on whole‐body homogenates from 28‐day‐old GALGLU‐HGLU versus GLU‐HGLU females, using the same experimental design as shown in Figure 4A. In total 504 polar lipid species were detected (Figure S5A), and partial least squares‐discriminant analysis (PLS‐DA) showed distinct lipidomic signatures between the two groups (Figure 5B). The relative abundance of 46 species was significantly lower, while the abundance of 43 species was significantly higher in the GALGLU‐HGLU, as shown using a Volcano plot with the significant lipid species color‐coded based on their main lipid classes (Student's *t*‐test *p* < 0.05 and VIP > 1; Figure 5C). All significant lipid species from each lipid subclass are shown in Figure 5D. We observed a systematically increased abundance of cardiolipin and lyso‐glycerophospholipid species, but decreased PC and PS species. Next, the lipidomic data were integrated into the simplified glycerophospholipid metabolism pathway adapted from KEGG databases (Figure 5E). Broad transcriptional regulation of key transcripts aligned with globally altered glycerophospholipid species abundance, with putative transcriptomic changes at the lipid main class level and lipidomic changes at the subclass level, although the complexity of lipid remodeling excluded simple transcript to lipid correlations.

### 3.5 Early‐Life GALGLU Diet Consumption Redirected PUFAs Toward Glycerophospholipids in Later‐Life

**FIGURE 6:**
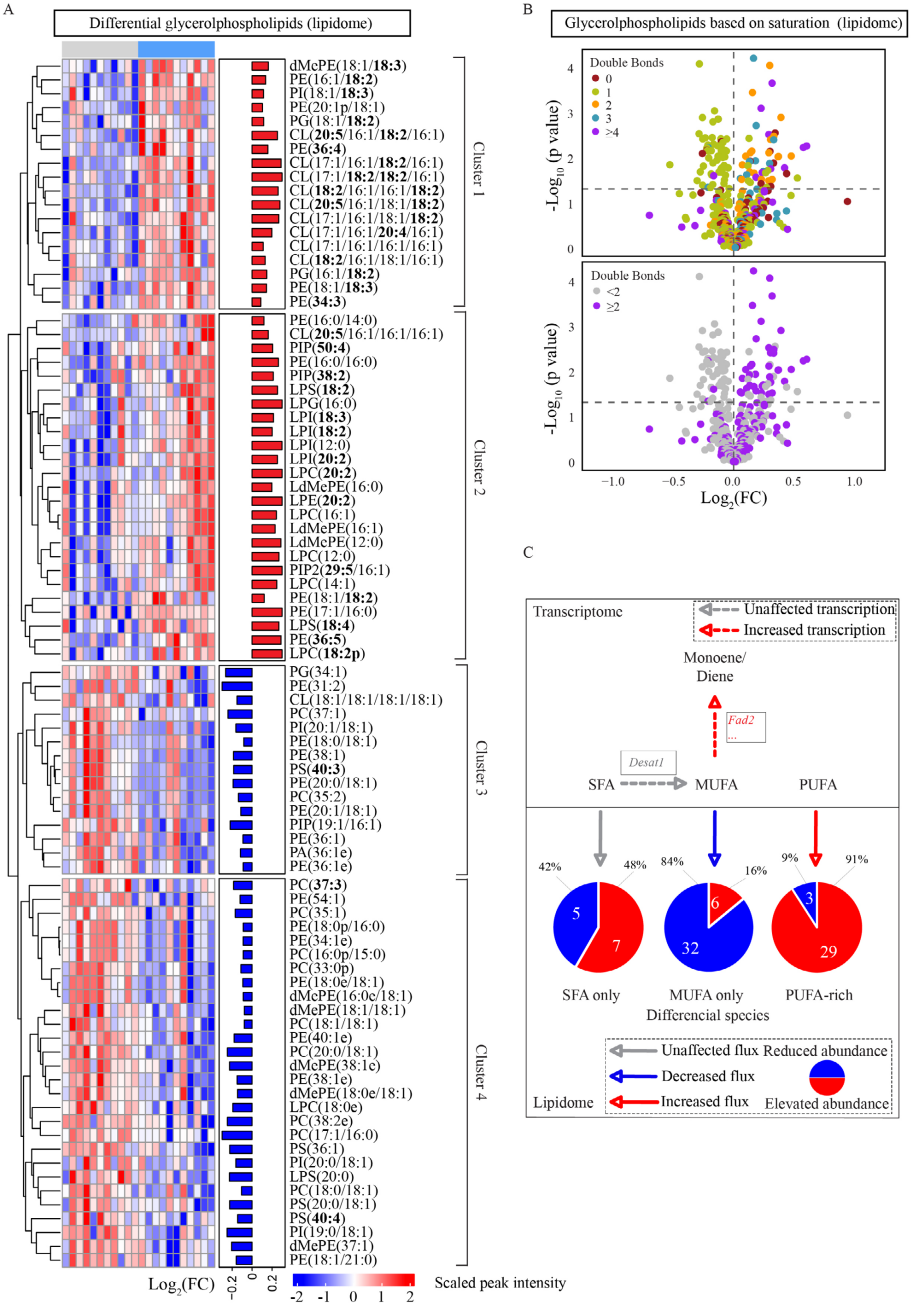
Early‐life co‐consumption of galactose and glucose (GALGLU), versus glucose alone (GLU), redirected polyunsaturated fatty acid toward glycerophospholipid synthesis in later‐life in female *Drosophila* maintained on a high‐glucose adult (HGLU) diet. Lipidomic data derive from the same experimental design outlined in Figure 4A (GALGLU‐HGLU vs. GLU‐HGLU). (A) Heatmap created by the scaled peak intensity of all significant lipid species. Lipid species were clustered into four categories. Polyunsaturated fatty acids were in bold. (B) Volcano plots of all detected glycerophospholipid species. Top, dots were colored by the number of double bonds in the glycerophospholipid species' most unsaturated fatty acyl chain. Bottom, all PUFA‐containing glycerophospholipid species were colored purple. (C) Integrated transcriptome and lipidome analysis. The transcriptional pathway is simplified from Figure 4D, where a complete list of involved transcripts is provided. The pie chart shows numbers and percentages of glycerophospholipid with different types of fatty acid (red sectors: higher abundance; blue sectors: lower abundance). SFA, saturated fatty acid; MUFA, monounsaturated fatty acid; PUFA, polyunsaturated fatty acid.

Hierarchical clustering of significant glycerophospholipids identified four distinct clusters (Figure 6A). Interestingly, clusters 1 and 2 were dominated by PUFA‐containing (highlighted in bold) glycerophospholipid species, such as PE(16:1/18:2) and PG(18:1/18:2). In contrast, clusters 3 and 4 were dominated by MUFA‐containing glycerophospholipid species, such as PG(34:1) and PE(18:0/18:1). Volcano plots further highlighted this finding: glycerophospholipids with higher abundance in GALGLU‐HGLU were enriched in PUFAs, while glycerophospholipid with lower abundance primarily contained either one or no double bonds (Figure 6B). A potential mechanistic explanation is described in Figure 6C. At the transcriptional level, MUFA synthesis remained constant between the two groups, while MUFA demand for CHC production increased. This created a relative shortage of MUFAs for membrane phospholipids, which is reflected in about 84% of significant MUFA‐containing glycerophospholipids decreasing. When MUFAs were in short supply, more PUFAs were incorporated into phospholipids. Therefore, about 91% significant PUFA‐containing glycerophospholipid species showed increased abundance.

Cumulatively, combining the lipidomics and transcriptomics data indicated that early‐life GALGLU consumption programmed higher levels of PUFA‐containing glycerophospholipid species in adulthood, concomitant with lower levels of MUFA‐containing glycerophospholipid species.

## 4 Discussion

In the present study, we tested whether natural co‐consumption of galactose and glucose (versus isocaloric glucose consumption) in larval early‐life period shapes the adult later‐life lifespan in *Drosophila melanogaster* . The GALGLU diet extended larval developmental time and reduced pupal energy expenditure. Strikingly, these early‐life developmental and metabolic adaptations were translated into long‐lasting, but sexually dimorphic, effects on adult lifespan: a shortened adult lifespan in males but protective effects against obesogenic diet‐induced attenuated lifespan in female flies. Transcriptome analyses of female flies identified metabolic programmed transcriptional regulation by GALGLU versus GLU of transcripts involved in VLCFA metabolism (e.g., *EloF* and *Spidey*), CHC biosynthesis (e.g., *Cyp4g1*), and glycerophospholipids metabolism (e.g., *Cds* and *Pect*), suggesting later‐life lipid remodeling by early‐life GALGLU diet. Indeed, lipidomic analyses in female flies showed systemic decreased glycerophospholipids saturation, characterized by increased PUFA‐containing glycerophospholipid species and decreased MUFA‐containing glycerophospholipid species, which aligned with and confirmed the identified transcriptional pathways.

While dietary restriction is a well‐established nutritional intervention that promotes longevity (Piper et al. 2011), it's less achievable in real‐world settings. Our data instead support a model in which early‐life carbohydrate quality induces long‐lasting metabolic programming effects that extend beyond caloric intake. Although early‐life consumption of both GALGLU (this study) and lactose (Morimoto et al. 2020) prolongs larval development, the physiological origin and their outcomes are distinct. The lactose‐induced reduction in pupal weight and energy reserves likely stems from a dietary restriction‐like phenotype due to the inability to digest lactose. In contrast, previous studies have confirmed that feeding rates remain comparable between galactose‐ and sucrose‐based diets (Jordens et al. 1999). Consistent with this, GALGLU consumption did not compromise pupal size, energy reserves, or survival rates, despite reducing energy expenditure. Moreover, the transcriptomic changes induced by GALGLU‐HGLU differs fundamentally from the typical dietary restriction–associated molecular signatures (Piper et al. 2011). Collectively, these observations indicate that while lactose is limited by its indigestibility in *Drosophila*, its constituent monosaccharides induce a distinct metabolic effect that is independent of dietary restriction.

Our transcriptome data suggested enhanced VLCFA and CHC biosynthesis, which are essential not only for desiccation resistance but also function in pheromone production in *Drosophila melanogaster* (Dembeck et al. 2015). VLCFAs play a conserved role in desiccation resistance, although they achieve this as components of ceramides or free fatty acids, rather than hydrocarbons, in mammals (Mizutani et al. 2009). Fewer unsaturated CHCs have also been shown to mediate larval yeast restriction‐induced adult lifespan extension in *Drosophila* (Stefana et al. 2017). Beyond these physiological effects, transcriptionally enhanced VLCFA and CHC biosynthesis may reduce the availability of cellular saturated fatty acid (SFA) and MUFA pools since SFAs and MUFAs, rather than PUFAs, are specifically consumed by these processes in *Drosophila* (Chertemps et al. 2006; Holze et al. 2021). Notably, *Fad2*, the only desaturase capable of introducing the second double bond required for diene CHC production (Chertemps et al. 2006; Dembeck et al. 2015), showed higher expression, whereas *Desat1*, the major MUFA‐producing desaturase (Dallerac et al. 2000; Murakami et al. 2017), was not different. This suggests increased MUFA demand without increased MUFA supply. Consistent with this idea, the lipidomic data showed reduced MUFA‐containing but increased PUFA‐containing glycerophospholipids. Our data support a model in which enhanced CHC biosynthesis, potentially mediated by *Fad2*, reduces MUFA availability for membrane lipids and thereby shifts adult membranes toward decreased saturation.

The saturation of the membrane lipids is a key determinant of membrane fluidity, which decreases during aging (Yu et al. 1992). Reduced membrane lipid saturation increases membrane fluidity, which may contribute to a longer lifespan (Papsdorf and Brunet 2019). On the other hand, reduced membrane lipid saturation and increased membrane lipid peroxidation are associated with aging since PUFAs are more sensitive to oxidative damage than MUFAs (Papsdorf and Brunet 2019). In our study, the GALGLU‐HGLU female flies showed higher expression of several transcripts involved in antioxidant synthesis, such as *Sod1* (*superoxide dismutase 1*) and *Gclc*/*m* (*Glutamate‐cysteine ligase catalytic/regulatory subunit*), which may suggest an improved antioxidant capacity, but this needs confirmatory functional testing. Meanwhile, the GALGLU‐HGLU females also showed a higher abundance of multiple cardiolipin species. In eukaryotes, cardiolipins are exclusively present in mitochondria. Cardiolipins are important mitochondrial inner membrane components that can influence mitochondrial bioenergetics (Horvath and Daum 2013). Therefore, increased cardiolipins potentially suggest a healthier mitochondrial inner membrane. Interestingly, the content of cardiolipins decreases with age, while restoring the cardiolipin content in old rats can rescue aging‐related dysfunctions (Petrosillo et al. 2009). Furthermore, we observed an increase in several lyso‐glycerophospholipid species, which are produced from diacyl‐glycerophospholipids via phospholipase A_2_ enzymes. Lyso‐glycerophospholipids serve as signaling molecules and play important roles in cellular signaling (Makide et al. 2009). The role of lyso‐glycerophospholipids in metabolic health is not well understood, and conflicting findings have been reported. For example, the concentrations of plasma lyso‐phosphatidylcholine and lyso‐phosphatidylethanolamine were negatively associated with body mass index and fasting glucose (Huynh et al. 2019), while positive associations of circulating lyso‐glycerophosphatidylcholine and metabolic diseases have also been reported (Law et al. 2019). Altogether, phospholipid metabolism remodeling characterized by increased PUFA‐containing glycerophospholipids (e.g., cardiolipin species) and lyso‐glycerophospholipids (e.g., lyso‐phosphatidylcholine species), in tissue in GALGLU‐HGLU versus GLU‐HGLU females, may represent a healthier lipid profile. This is concomitant with an increased lifespan, but whether this mediates the observed extended lifespan remains to be further elucidated.

The sexual dimorphism in developmental metabolic programming studies has been well noted, and the sexually dimorphic effects may depend on the specific context (Dearden et al. 2018). Overall, it seems that the metabolic health of females may be more susceptible to early‐life interventions. For instance, female embryos exposed to the Dutch famine during gestation had a higher body mass index and increased adiposity in later life (Stein et al. 2007). Reduced carbohydrate intake in early life showed protective effects on type 2 diabetes development in later life, and these effects are more pronounced in women than in men (Gracner et al.). Our findings align with previous results for sex‐specific long‐term benefits of early‐life galactose in mice, showing that isocaloric replacement of glucose with galactose in the postweaning diet reduced later‐life adiposity in female, but not male, mice under obesogenic nutritional conditions (Bouwman, Fernández‐Calleja, et al. 2019). Dimorphic nutritional responses have also been observed in *Drosophila*. In *Drosophila melanogaster* , male flies are more tolerant of excess carbohydrate‐induced shortened lifespan than female flies (Chandegra et al. 2017). Together, this underlines the existence of a sexual dimorphic response to metabolic substrates in *Drosophila*. The higher expression of *Fad2* in GALGLU‐HGLU females raises the possibility that *Fad2*‐dependent CHC remodeling contributes to the sexually dimorphic lifespan effects observed in our study, since *Fad2* expression is female‐biased and plays a central role in shaping sex‐specific CHC profiles. Nevertheless, the mechanisms underlying larval GALGLU diet‐induced sexually dimorphic effects on adult lifespan remain to be elucidated.

The metabolic impact of GALGLU feeding on larvae and pupae, and its relationship to adult lifespan and metabolic alterations, is a fundamental topic warranting in‐depth investigation. The current data provide a foundation to further study this. Outcomes of such studies may be of environmental importance, since *Drosophila* can feed on nutrient sources with different amounts of galactose (Mansourian et al. 2018). Translation to other organisms, like mice and humans, of the long‐term beneficial effects of galactose on adult lifespan and its effect on glycerophospholipid saturation profiles in female *Drosophila* warrants caution. Nevertheless, our results align with previous findings in mice, where postweaning dietary galactose improves metabolic health in young adult females (Bouwman, Fernández‐Calleja, et al. 2019; Sun et al. 2022), and with a prior study showing that early‐life galactose, versus glucose, promotes healthy aging in yeast (Horkai et al. 2023), and further support the view that galactose is an overlooked carbohydrate. Moreover, by highlighting the programming effects of carbohydrate quality during early life, our findings broaden the concept of nutritional programming, which mainly focused on macronutrient quantity or malnutrition (Hoffman et al. 2021). Currently, the European Food Safety Authority guideline in Europe prohibits galactose and other free carbohydrates in infant formulae (EFSA Panel on Dietetic Products, Nutrition and Allergies 2014), whereas the United States Food and Drug Administration's regulations do not specify the source of carbohydrate that can be used in an infant formula (DiMaggio et al. 2024). Therefore, more scientific evidence is needed to determine the optimal composition.

Concluding, we newly observed that early‐life galactose and glucose co‐consumption exerted sexual dimorphic effects on adult lifespan in *Drosophila*. While detrimental to males, it protected females specifically by counteracting the life‐shortening effects of an obesogenic adult diet. This protection may be mediated by a healthier lipid profile, characterized by the redirection of PUFAs toward glycerophospholipid synthesis.

## Author Contributions

**Jaap Keijer**, **Evert M. van Schothorst**, **Jing Tang**, and **Steven Driever:** resources. **Jaap Keijer** and **Evert M. van Schothorst:** conceptualization and supervision study. **Peixin Sun**, **Shiying Shao**, **Robin W. Creemers**, and **Steven Driever:** execution. **Peixin Sun**, **Anna F. Bekebrede**, **Jaap Keijer**, and **Evert M. van Schothorst:** data analysis. **Peixin Sun** and **Evert M. van Schothorst:** original draft. **Peixin Sun**, **Jaap Keijer**, and **Evert M. van Schothorst:** data interpretation. **Peixin Sun**, **Jaap Keijer**, and **Evert M. van Schothorst:** writing, review, and editing. All authors have read and agreed to the published version of the manuscript.

## Funding

Peixin Sun is funded by the China Scholarship Council (Grant 202003250074).

## Ethics Statement

The authors have nothing to report.

## Conflicts of Interest

The authors declare no conflicts of interest.

## Supporting information


**Data S1:** acel70429‐sup‐0001‐Supinfo.pdf.

**Data S2:** acel70429‐sup‐0002‐Supinfo2.docx.

## Data Availability

RNA‐seq data files are openly available in Gene Expression Omnibus (GEO), GSE297977. All other data that support the findings of this study are available from the corresponding author upon reasonable request.
