# Supplementary material for: Lactose‐Derived Carbohydrates Induce Sexually Dimorphic Nutritional Programming Effects on Lifespan in Drosophila melanogaster
Source: Aging Cell. 2026 Feb 26;25(3):e70429. doi: 10.1111/acel.70429 (PMC12938501; doi:10.1111/acel.70429)
Supplement: Supplementary file 1 — Data S1: acel70429‐sup‐0001‐Supinfo.pdf. [file ACEL-25-e70429-s001.pdf]

AGING CELL AUTHOR CHECKLIST. ***Authors should submit this checklist together with their manuscript. Please ensure that you have read the Author Guidelines in detail before submission.***

**Title**	Lactose-derived carbohydrates induce sexually dimorphic nutritional programming effects on lifespan in Drosophila melanogaster
**Authors**	Peixin Sun, Shiying Shao, Robin W. Creemers, Anna F. Bekebrede, Jing Tang, Steven Driever, Jaap Keijer, and Evert M. van Schothorst
**Manuscript Type**	Research articles
**Total Character Count (including spaces)[1]**	41,388
**Word count of Summary[2]**	231
**Number of papers cited in the References[3]**	45
**Listing of all Tables (Table1, Table 2 etc)[4]**	Table 1

**Figure specifications (please complete one row per figure)[5]** ***Figure no.***	Colour ***(yes/no)***	Greyscale ***(yes/no)***	Black and white ***(yes/no)***	Single column (80mm) ***(yes/no)***	Double column (180mm) ***(yes/no)***	Size of figure at full scale (mm x mm) ***(insert details)***	Smallest font size used in the figure at full scale (minimum 6pt) ***(insert details)***
1	yes	no	no	yes	no	210*279	12
2	yes	no	no	yes	no	210*216	12
3	yes	no	no	yes	no	210*216	12
4	yes	no	no	yes	no	210*279	10
5	yes	no	no	yes	no	210*279	10
6	yes	no	no	yes	no	210*279	10

[1] The maximum character count allowed is 50,000 (incl. spaces) for Primary Research Papers and Reviews, 10,000 for Short Takes.

[2] Summary should not exceed 250 words.

[3] Primary Research Papers can contain a maximum of two tables. If more are needed they should replace some of the Figures or can be placed in the Supporting Information.

[4] A maximum of 45 references is allowed for Primary Research Papers and 20 references for Short Takes.

[5] A Primary Research Paper may contain up to 6 figures and a Short Take up to 2 figures. Authors are encouraged to provide figures in the size they are to appear in the journal and at the specifications given.
