# Supplementary material for: Lactose‐Derived Carbohydrates Induce Sexually Dimorphic Nutritional Programming Effects on Lifespan in Drosophila melanogaster
Source: Aging Cell. 2026 Feb 26;25(3):e70429. doi: 10.1111/acel.70429 (PMC12938501; doi:10.1111/acel.70429)
Supplement: Supplementary file 2 — Data S2: acel70429‐sup‐0002‐Supinfo2.docx. [file ACEL-25-e70429-s002.docx]

**Supplementary methods**

**Synchronized egg collection**
Approximately 400 adult flies, a mixture of males and females aged 4 to 12 days post-eclosion, were placed in one embryo collection cage (59-101, Genesee Scientific, San Diego, USA) (Linford, Bilgir, Ro, & Pletcher, 2013). The cage was closed containing a petri dish with 20 mL GLU diet and yeast paste (a mixture of water and yeast, to stimulate the egg production) for a 24-hour acclimatization period. Following this, the petri dish was replaced by a new one with fresh GLU diet and yeast paste, and the flies were allowed to mate and produce synchronized eggs over a 5-hour period. Eggs were then collected with phosphate-buffered saline (PBS) and were randomly seeded at a density of 80 to 120 eggs per vial containing 6 mL of the specified diet.

Following this protocol, approximately 1,200 eggs can be obtained per collection cage. Depending on the experimental requirements, multiple collection cages were prepared to ensure sufficient numbers of pupae or flies.

**Citrate synthase content**

Groups of 5 pupae were homogenized in 150 μL lysis buffer containing 50 mM Tris-HCl pH 7.4, 150 mM NaCl, 1% Triton X-100, 1% glycerol,1 mM Ethylenediaminetetraacetic acid, 2 μM trichostatin A, 10 mM nicotinamide and one tablet per 10 mL of both protease (4693132001, Merk) and phosphatase inhibitor cocktail (4906845001, Merk). The homogenates were then submitted to a freeze-thaw cycle (−80 °C freezing, 4 °C thawing), after which the samples were centrifuged at 10,000 x *g* and 4 °C to remove cell debris. The protein content was determined using the DC protein assay reagent (5000116, Bio-Rad, Veenendaal, The Netherlands). Samples, acetyl coenzyme A and 5,5’-Dithiobis-(2-nitrobenzoic acid) (DTNB) were first added to measure the baseline absorbance at 412 nm. Then, oxaloacetate was added to initiate the reaction, changes in absorbance at 412 nm were monitored for 20 minutes with a measurement interval of 30 seconds, using a Synergy HT Multi-detection microplate reader (BioTek Instruments, Inc., Winooski, USA). The activity was calculated using the equation:

$$Citrate synthase content (\mu mol/min/mg protein)= \frac{Delta A412 \times V}{\varepsilon\times L \times P}$$

with parameters Delta A412: the changes in absorbance at 412 nm after 1.5 minutes of the reaction; V: reaction volume, 0.2 (mL); ε: 13.6 (mM^-1^ cm^-1^), the extinction coefficient of 5,5'-Dithiobis-(2-nitrobenzoic acid) at 412 nm; L: the pathlength, 0.552 (cm); P: the protein input (mg).

**Adult body weight measurement**

At eclosion, flies from each vial were first anesthetized by CO_2_, and the male and female flies were separated. For each vial, the total body weight of 20 flies per sex was measured, and the mean individual body weight was calculated by dividing the total weight by 20. Thus, one data point represents the average of 20 flies per sex per vial.

After reaching sexual maturation, the female and male flies were housed separated on the HGLU diet for 28 days. The body weight of GALGLU-HGLU and GLU-HGLU was measured at 7, 14, 21, and 28 days after eclosion. One data point represents the average of 10-20 flies per sex per vial.

**Whole-body triacylglycerol measurement**

For pupae, five pupae per sample were homogenized in 500 µl 0.05% PBST (PBS with 0.05% Tween 20). For adult flies, four flies per sample were homogenized in 150 (for males) or 300 µl (for females) 0.05% PBST. The homogenate was aliquoted for either whole-body TAG or protein measurements. The homogenate for whole-body TAG content measurement was heated at 65°C for 5 minutes, followed by the TAG measurements. The homogenate for protein measurements was diluted 5 times, and the protein content was measured by DC protein assay reagent (Bio-Rad) according to the manual. TAG and protein assays were performed in duplicate, and all the samples with a coefficient of variation (CV%) higher than 20% were excluded from analyses. TAG assay was performed in duplicate, and all the samples with a coefficient of variation (CV%) higher than 20% were excluded from analyses, resulting in 2 and 3 data points being excluded from GLU and GALGLU groups, respectively.

**Transcriptomics**

Total RNA was extracted from pooled abdominal carcasses using Invitrogen Trizol reagent (Thermo Fisher Scientific, Schwerte, Germany), according to the manufacturer’s instructions. RNA integrity was verified using the Agilent 2100 Tapestation (Agilent Technologies Inc., Santa Clara, USA); all samples had an RNA integrity number above 7. RNA preparation, library construction, sequencing on the DNB-sequencing platform, and read clean-up were performed at Beijing Genomics Institute (BGI, Hong Kong, People’s Republic of China). Quality check of the clean reads was performed using FASTQC ("Babraham Bioinformatics. FastQC: a quality control tool for high throughput sequence data, https://www.bioinformatics.babraham.ac.uk/projects/fastqc/,"), and high-quality sequencing reads were obtained, with a minimum of 91.1 % of bases at Q‑score ≥ 30 (FASTQC, version 0.12.1). Reads were aligned to the *Drosophila melanogaster* genome (BDGP6.46) using STAR2.7 (Dobin et al., 2013), and counts were quantified using HTSeq (Anders, Pyl, & Huber, 2015). The average sequencing depth was 24M paired-end reads, of which at least 91.4% were uniquely mapped. RNA-sequencing was performed, and data pre-processing was conducted in a blinded manner.

**Lipidomics**

Sample preparation and lipid extraction

Samples were homogenized in 800 µL of pre-chilled dichloromethane/methanol precipitation agent (3:1, v/v) containing 10 µL of prepared internal standard using TissueLyser for 5 min and ice-bath sonication for 10 min. After overnight incubation at -20°C, samples were centrifuged at 25,000 × *g* (4°C) for 15 min. A 600 µL aliquot of supernatant was vacuum-dried and reconstituted in 600 µL lipid resuspension solution (isopropanol:acetonitrile:water = 2:1:1, v/v/v) and vortexed for 10 min. After 10 min of ice-bath sonication, the reconstituted samples were centrifuged at 25,000 × *g* (4°C) for 15 min, and the supernatant was collected for the subsequent analysis. Quality control samples were prepared by pooling 20 µL from each sample supernatant and were interspersed in the sample measurements to evaluate the stability of the system.

Chromatographic conditions

Lipids were separated using the Waters 2777c Ultra Performance Liquid Chromatography (Waters, Milford, USA) with a CSH C18 column(1.7 μm; 2.1*100 mm). At positive ion mode with mobile phase A consisting of 60% acetonitrile in water + 10mM ammonium formate + 0.1% formic acid and mobile phase B consisting of 90% isopropanol + 10% acetonitrile + 10mM ammonium formate + 0.1% formic acid. The column temperature was maintained at 55 °C. The gradient conditions were as follows: 40%~43% B over 0~2 min, 43%~50% B over 2~2.1 min, 50%~54% B over 2.1~7 min, 54%~70% B over 7~7.1 min, 70%~99% B over 7.1~13 min, 99%~40% B over 13~13.1 min, held constant at 99%~40% B over 13.1~15 min and washed with 40% B over 13.1-15 min. The flow rate was 0.4 mL/min, and the injection volume was 5 μL.

Mass spectrometry conditions

The primary and secondary mass spectrometry data were obtained using the Q Exactive HF High Resolution Mass Spectrometer (Thermo Fisher Scientific, Waltham, USA). The full scan range was 70‒1,050 m/z with a resolution of 120,000, and the automatic gain control (AGC) target for MS acquisitions was set to 3e6 with a maximum ion injection time of 100 ms. The top 3 precursors were selected for subsequent MSMS fragmentation with a maximum ion injection time of 50 ms and a resolution of 30,000, and the AGC was 1e5. The stepped normalized collision energy was set to 15, 30, and 45 eV. ESI parameters were set as: Sheath gas flow rate was 40, Aux gas flow rate was 10, positive-ion mode Spray voltage(|KV|) was 3.80, negative-ion mode Spray voltage(|KV|) was 3.20, Capillary temperature was 320°C. Aux gas heater temperature was 350°C. Positive ion mode adduct form: [M+H]+, [M+NH4]+, [M+Na]+. Negative ion mode adduct form: [M-H]-, [M-2H]-, [M+HCOO]-. Retention time deviation:0.1min. The mass spectrometer data were analyzed in lipidsearch v.4.1 (Thermo Fisher Scientific, USA) software. Species with more than 50% missing values in QC samples and more than 80% missing values were filtered. K-Nearest Neighbor Algorithm was applied to fill in missing values. Probabilistic Quotient Normalization was applied to normalize the data. Lipidomics was performed, and data pre-processing was conducted in a blinded manner.

**Supplementary data**


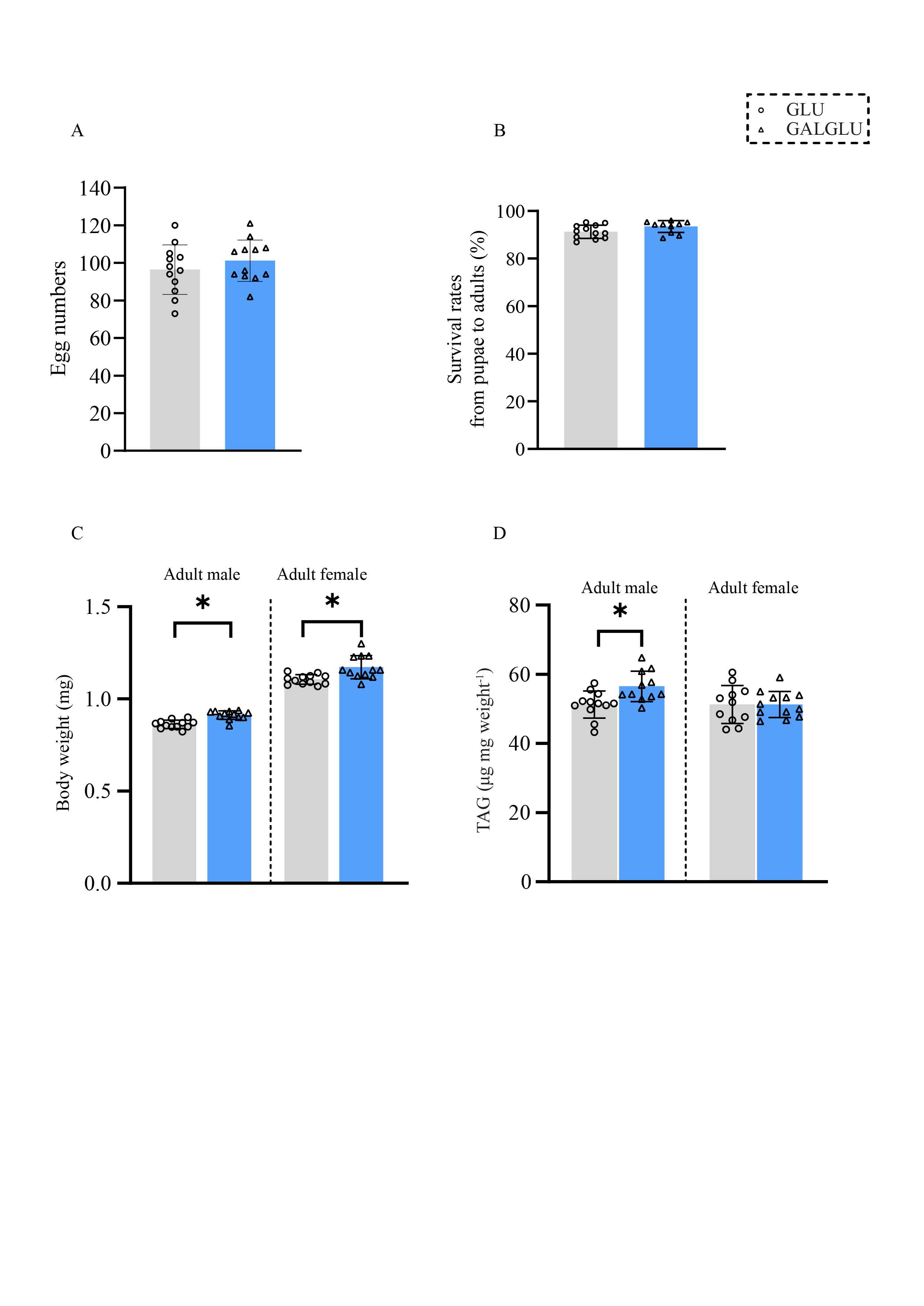


Figure S1: Co-consumption of galactose and glucose (GALGLU) compared to glucose (GLU) in early-life (larvae) affected body weight and whole-body triacylglycerol (TAG) content of eclosed adult flies, without affecting the eclosion rate. (A) The seeded egg numbers (n=12 samples per group). (B) Survival rates from pupae to adult flies (n=10-12 samples per group, with 80-120 eggs per sample). (C) Body weight of male flies and female flies; values represent the average weight per fly, determined by measuring total weight of 20 flies (n = 12 samples per group, with 20 flies per sample). (D) Whole-body TAG content of male flies and female flies; TAG content was normalized by body weight (n = 11-12 samples per group with 4 flies per sample).


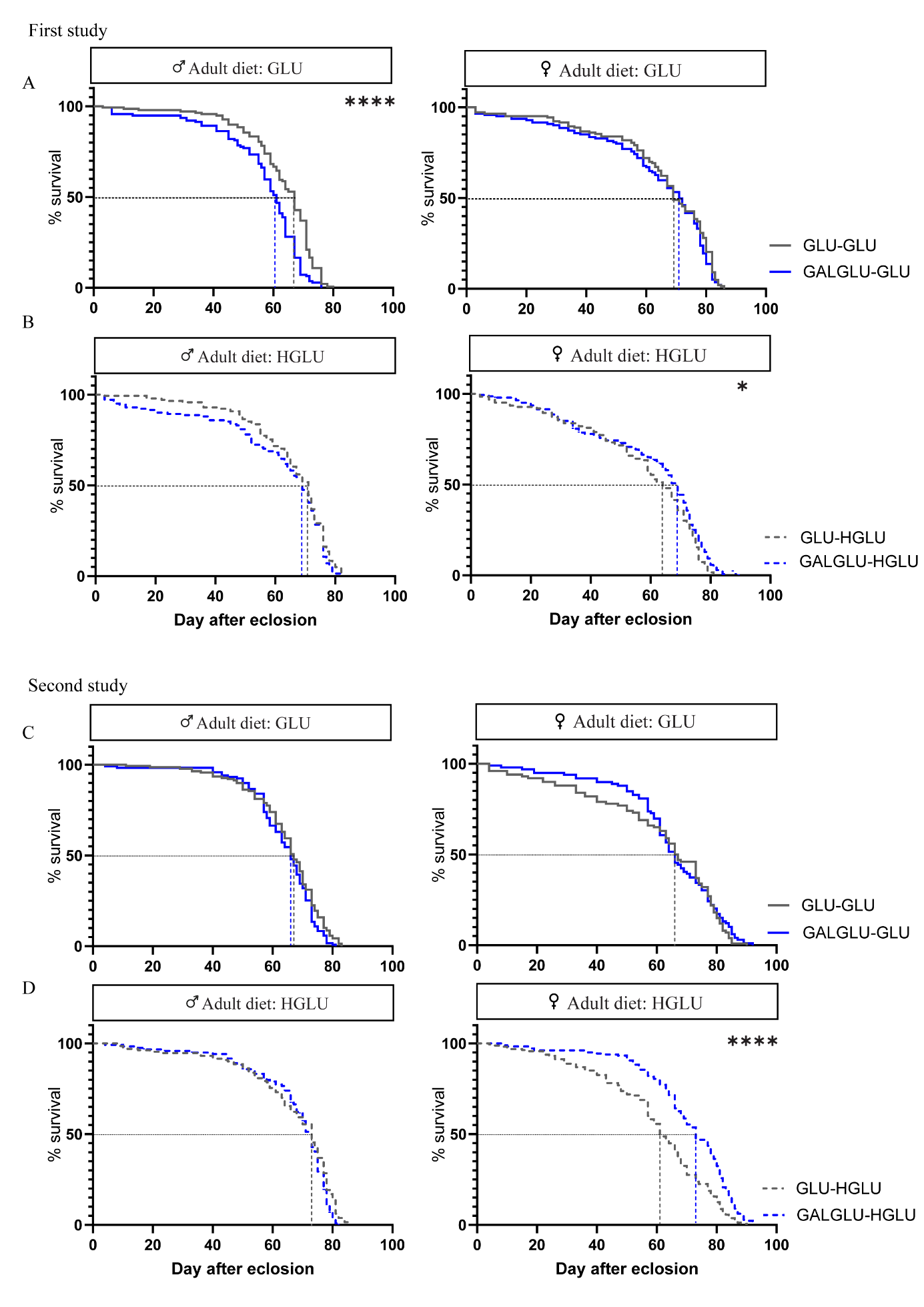


Figure S2: Co-consumption of galactose and glucose (GALGLU) compared to glucose (GLU) in early-life (larvae) regulated adult lifespan upon feeding either GLU or high-glucose (HGLU) adult diet in *Drosophila*. The experiments were repeated twice. (A) Lifespan curves of male flies (n = 138 flies in GLU-GLU, n = 139 flies in GALGLU-GLU) and female flies (n = 143 flies in GLU-GLU, n = 139 flies in GALGLU-GLU) when maintained on GLU diet in the first study. (B) Lifespan curves of male flies (n = 141 flies in GLU-HGLU, n = 141 flies in GALGLU-HGLU) and female flies (n = 123 flies in GLU-HGLU, n = 140 flies in GALGLU-HGLU) when maintained on HGLU diet in the first study. (C) Lifespan curves of male flies (n = 138 flies in GLU-GLU, n = 119 flies in GALGLU-GLU) and female flies (n = 100 flies in GLU-GLU, n = 99 flies in GALGLU-GLU) when maintained on GLU diet in the second study. (D) Lifespan curves of male flies (n = 130 flies in GLU-HGLU, n = 119 flies in GALGLU-HGLU) and female flies (n = 160 flies in GLU-HGLU, n = 179 flies in GALGLU-HGLU) when maintained on HGLU diet in the second study. Statistical analysis was performed by the log-rank test (∗*p* < 0.05; ∗∗∗∗*p* < 0.0001).


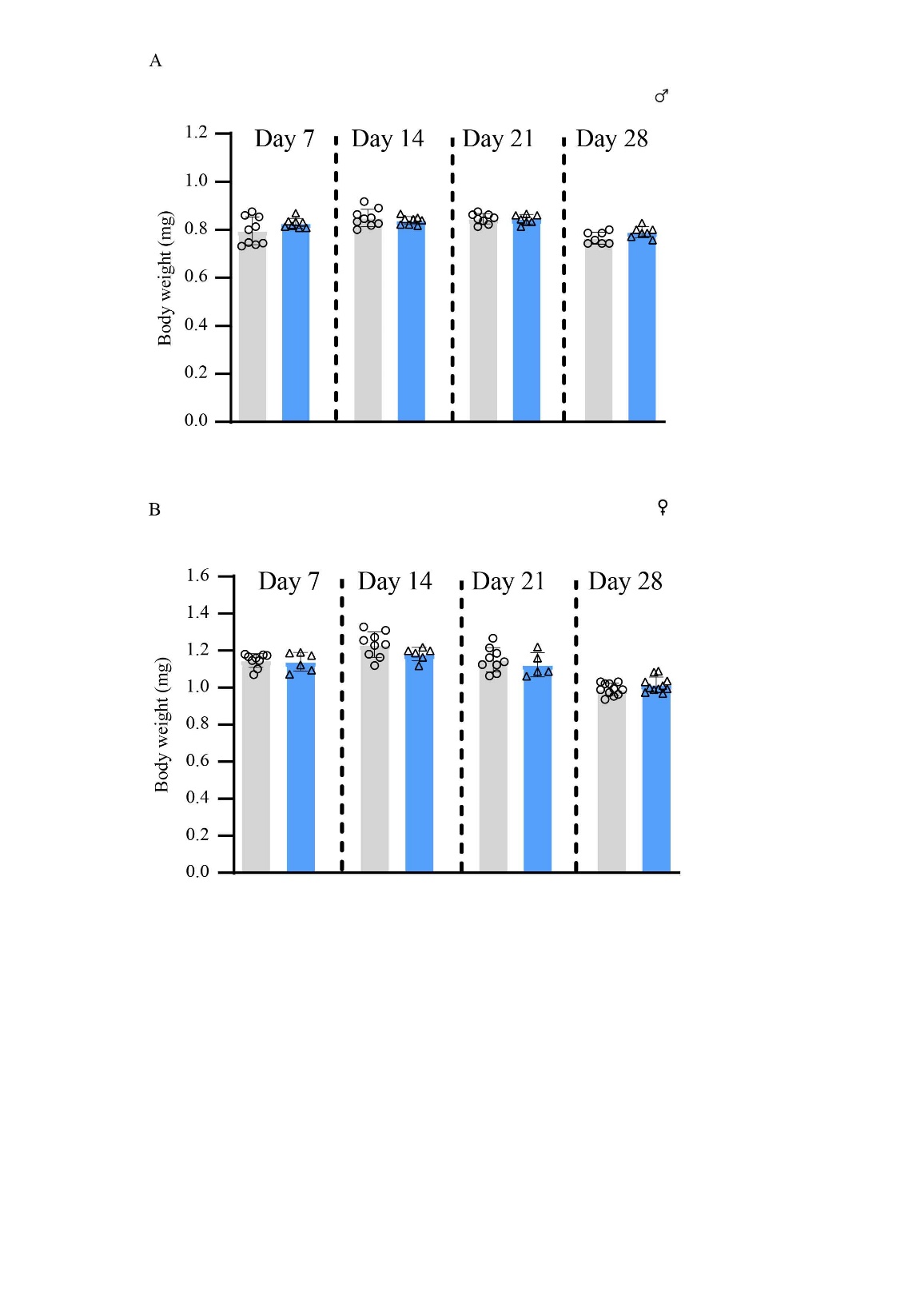


Figure S3: Co-consumption of galactose and glucose (GALGLU) compared to glucose (GLU) in early-life (larvae) did not affect adult body weight upon feeding either high-glucose (HGLU) adult diet in *Drosophila*. (A) Body weight of male flies at 7, 14, 21 and 28 days post-eclosion; (B) Body weight of female flies at 7, 14, 21 and 28 days post-eclosion. Values represent the average weight per fly, determined by measuring total weight of 10-20 flies (n = 5-11 samples per group, with 10-20 flies per sample).


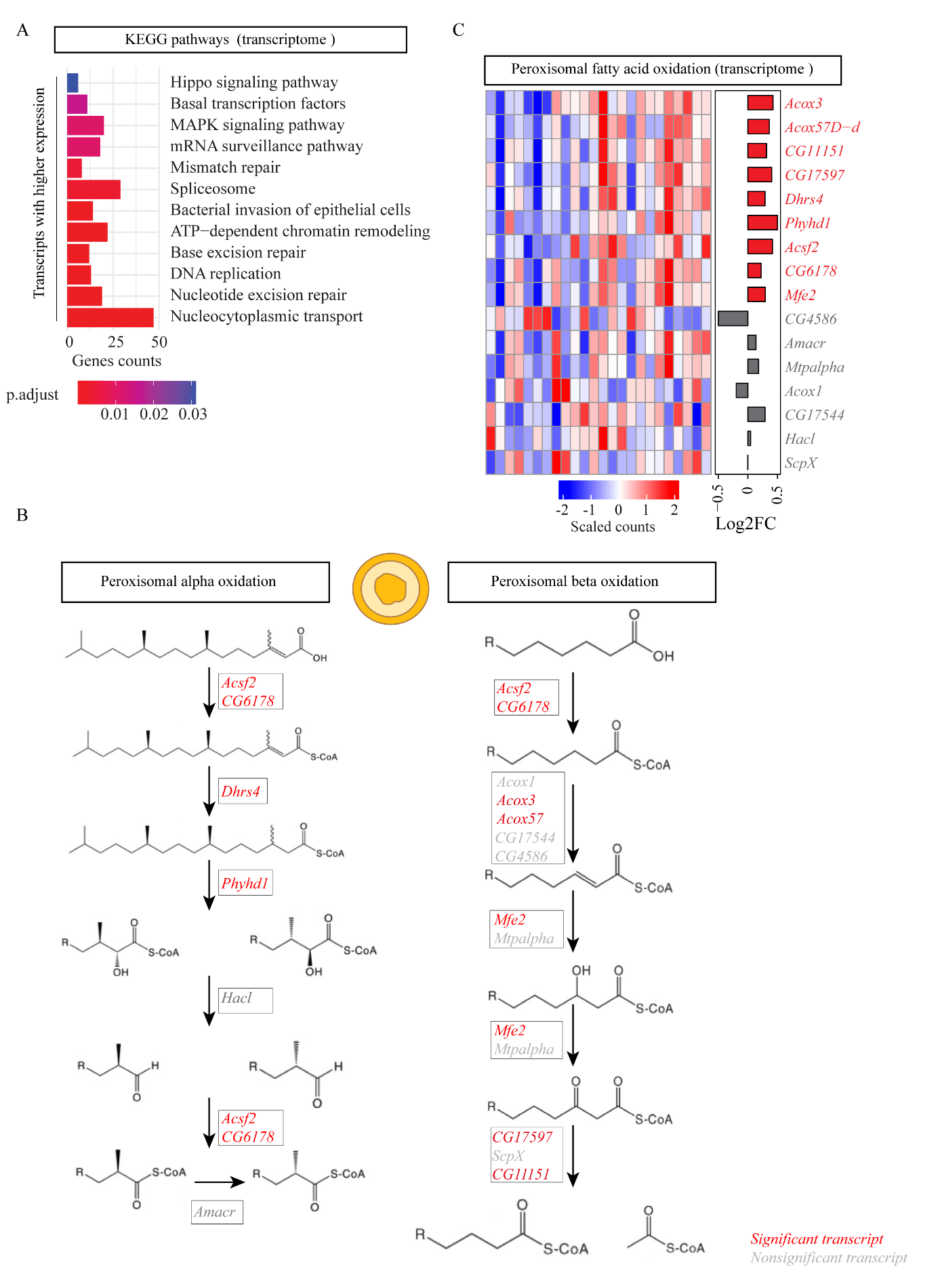


Figure S4: Transcriptomics in abdominal carcasses (primarily composed of the fat body and oenocytes) of GALGLU-HGLU (co-consumption of galactose and glucose during larvae and high-glucose during adult) versus GLU-HGLU (glucose during larvae and high-glucose during adult) female flies. (A) Kyoto Encyclopedia of Genes and Genomes pathways enriched by transcripts with significantly lower expression. (B) Peroxisomal fatty acid alpha and bata-oxidation, adapted from (Faust, Verma, Peng, & McNew, 2012). Transcripts are represented as rectangles, red: transcripts with higher expression; grey: unchanged transcripts. (C) Heatmap created by the scaled counts of key transcripts involved in peroxisomal fatty acid alpha and beta oxidation, with the log2 (fold change) of GALGLU-HGLU/GLU-HGLU. *Acox3: Acyl-CoA oxidase 3; Acox 57D-d:* [*acyl-Coenzyme A oxidase at 57D*](https://flybase.org/reports/FBgn0034628) *distal; Dhrs4: Dehydrogenase/reductase 4; Phyhd1: Phytanoyl-CoA dioxygenase domain containing 1; Acsf2: Acyl-CoA synthetase family member 2; Mfe2: peroxisomal Multifunctional enzyme type 2; Amacr: Alpha-methylacyl-CoA racemase; Acox1: Acyl-CoA oxidase 1; Hacl: 2-hydroxyacyl-CoA lyase; ScpX:Sterol carrier protein X-related.*


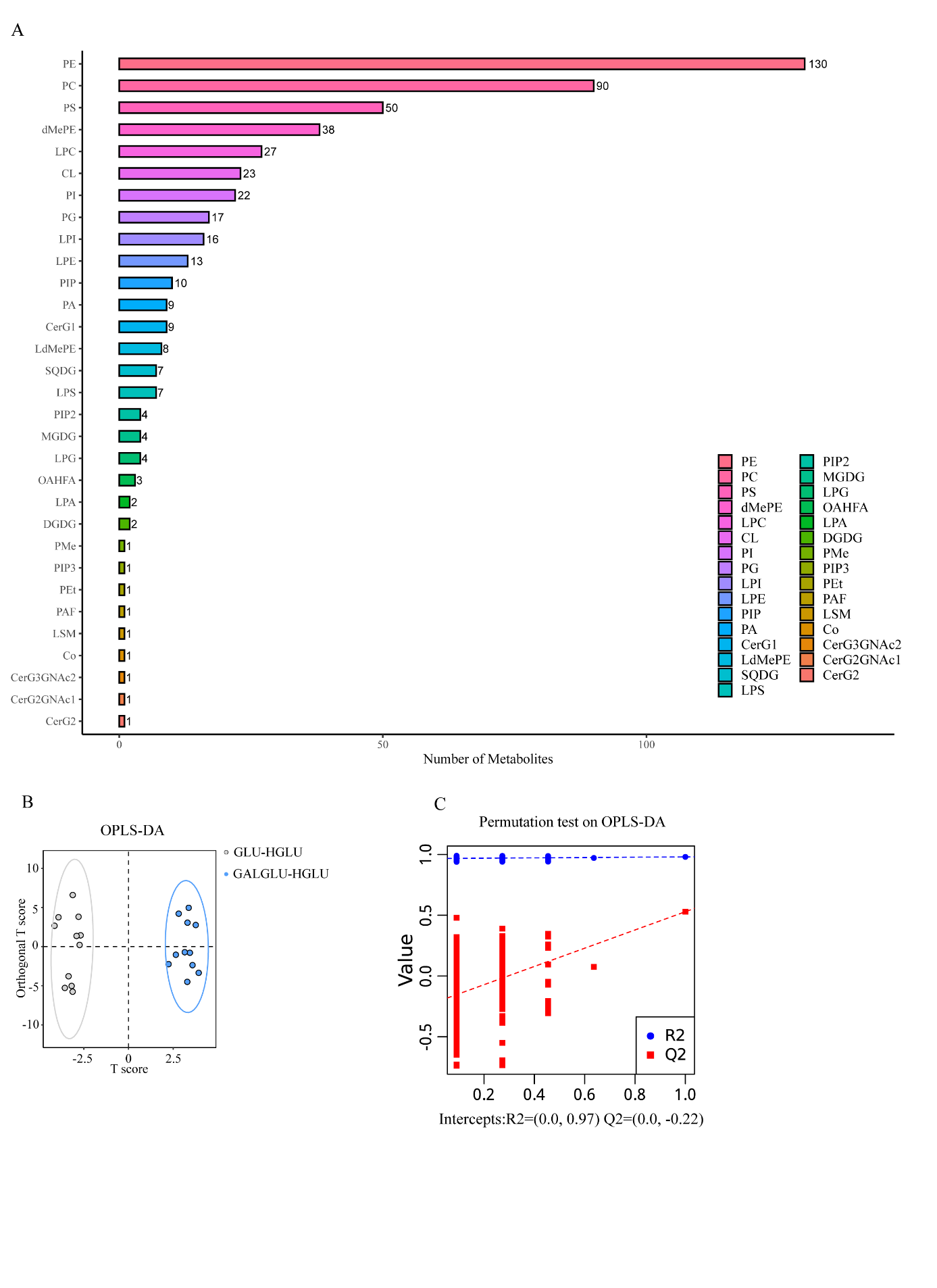


Figure S5: Whole-body lipidomics was performed on (co-consumption of galactose and glucose during larvae and high-glucose during adult) versus GLU-HGLU (glucose during larvae and high-glucose during adult) females 28 days after eclosion. (A) total identified lipid species under each sub class. The lipid classes and abbreviation can be found in Table S4 (B) orthogonal partial least squares-discriminant analysis model (OPLS-DA). (C) Permutation test on the OPLS-DA model.

Table S1: Lifespan parameters in Figure 2

| Sex | Early-life diets | Later-life diets | Number of flies | Median survival | Average survival | Maximum survival | Log-rank test | Hazard ratios |
| --- | --- | --- | --- | --- | --- | --- | --- | --- |
| Male | GLU | GLU | 276 | 67 | 63 | 79 | <0.0001 | GALGLU-GLU/GLU-GLU: 1.43 (95% CI: 1.2 - 1.7) |
|  | GALGLU | GLU | 258 | 63 | 60 | 76 |  |  |
|  | GLU | HGLU | 271 | 71 | 65 | 82 | 0.016 | GALGLU-HGLU/GLU-HGLU: 1.26 (95% CI: 1.02 - 1.44) |
|  | GALGLU | HGLU | 260 | 71 | 63 | 80 |  |  |
| Female | GLU | GLU | 243 | 69 | 63 | 84 | 0.91 | GALGLU-GLU/GLU-GLU: 1.01 (95% CI: 0.84 - 1.2) |
|  | GALGLU | GLU | 238 | 69 | 63 | 85 |  |  |
|  | GLU | HGLU | 283 | 62 | 58 | 83 | <0.0001 | GALGLU-HGLU/GLU-HGLU: 0.63 (95% CI: 0.54 - 0.75) |
|  | GALGLU | HGLU | 319 | 71 | 66 | 87 |  |  |

During the early-life (larval period), larvae were fed a standard glucose diet (GLU) or a GALGLU diet containing galactose and glucose in a 1:1 ratio. During the later-life (adult) period, the flies were fed a GLU or a high-glucose (HGLU) diet. Groups are denoted by early diet-later diet (e.g., GLU-HGLU means Drosophila fed GLU diet during larval period and HGLU diet in adulthood). Median survival, maximum survival and average lifespan are given as days and values and are calculated for the entire population within each experimental condition.

Table S2: Lifespan parameters in Figure 3

| Sex | Early-life diets | Later-life diets | Number of flies | Median survival | Average survival | Maximum survival | Log-rank test | Hazard ratios |
| --- | --- | --- | --- | --- | --- | --- | --- | --- |
| Male | GLU | SUC | 126 | 70 | 65 | 82 | GALGLU-SUC vs GLU-SUC: 0.018 GALGLU-SUC vs SUC-SUC: 0.12 | GALGLU-SUC/GLU-SUC:1.34 (95% CI: 1.02- 1.75)  GALGLU-SUC/SUC-SUC:0.83 (95% CI: 0.64- 1) |
|  | SUC | SUC | 138 | 63 | 62 | 78 |  |  |
|  | GALGLU | SUC | 100 | 66 | 65 | 82 |  |  |
| Female | GLU | SUC | 103 | 66 | 62 | 87 | GALGLU-SUC vs GLU-SUC: 0.016 GALGLU-SUC vs SUC-SUC: <0.0001 | GALGLU-SUC/GLU-SUC:0.73 (95% CI: 0.56- 0.97)  GALGLU-SUC/SUC-SUC:0.62 (95% CI: 0.48- 0.8) |
|  | SUC | SUC | 135 | 59 | 58 | 86 |  |  |
|  | GALGLU | SUC | 104 | 78 | 68 | 88 |  |  |
| Male | GLU | HSUC | 108 | 69 | 63 | 81 | GALGLU-HSUC vs GLU-HSUC: 0.5  GALGLU-HSUC vs SUC-SUC: 0.17 | GALGLU-HSUC/GLU-HSUC:1.1 (95% CI: 0.83- 1.44)  GALGLU-HSUC/SUC-HSUC:1.18 (95% CI: 0.9- 1.5) |
|  | SUC | HSUC | 127 | 67 | 65 | 82 |  |  |
|  | GALGLU | HSUC | 98 | 69 | 64 | 80 |  |  |
| Female | GLU | HSUC | 101 | 67 | 63 | 81 | GALGLU-HSUC vs GLU-HSUC 0.04 GALGLU-HSUC vs SUC-SUC 0.0003 | GALGLU-HSUC/GLU-HSUC:0.76 (95% CI: 0.58- 1.01)  GALGLU-HSUC/SUC-HSUC:0.65 (95% CI: 0.5- 0.84) |
|  | SUC | HSUC | 140 | 71 | 65 | 82 |  |  |
|  | GALGLU | HSUC | 102 | 79 | 64 | 80 |  |  |

During the early-life (larval period), larvae were fed a standard glucose diet (GLU) or a GALGLU diet containing galactose and glucose in a 1:1 ratio or a standard sucrose diet (SUC). During the later-life (adult period), the flies were fed a SUC or a high-SUC (HSUC) diet. Groups are denoted by early diet-later diet (e.g., GLU-SUC means Drosophila fed GLU diet during larval period and SUC diet in adulthood). Median survival, maximum survival and average lifespan are given as days and values and are calculated for the entire population within each experimental condition.

Table S3: Primer sequences and annealing temperatures in real-time quantitative polymerase chain reaction

| Gene | Sequence (5’ -> 3’)  Forward primer | Sequence (5’ -> 3’)  Reverse primer | Product length (bp) | Annealing Temperature (^o^C) |
| --- | --- | --- | --- | --- |
| *FASN3* | CAAAAGCCCCAGTTGTTGTGT | GCTTCCCCAAGAACCATTGC | 218 | 58 |
| *Bcat* | CTGATCGGCATTGATCCCACA | CATGGTGCCCACTTCGGTTA | 288 | 58 |
| *eloF* | AATCATGGAGCACCGAAAGC | GACAAACAGGAAGTGAACTCCTAAA | 115 | 58 |
| *Spidey* | GGCCAAGGAGATAGGCGATAA | ACCGAGTGGATATTGGCAGC | 231 | 58 |
| *Fad2* | CAACGGTCGTGCTCTTTTGG | TTGAGCGGTGTATTCGCCTT | 109 | 58 |
| *Cyp4g1* | ACGTCCAGACATCTACCCCA | TGACGCTTCTCCAACGAGAC | 279 | 58 |
| *ATPsynC* (Ref) | GGGTTCGTTCGAAAGCCGT | AACGGGGGTTGTGTTCTGAG | 212 | 58 |
| *Cyp1* (Ref) | TGATTCGACAGTTTCGGCACA | GTTGTCGGCGGTCATATCAAA | 209 | 58 |

Table S4: Lipid class and abbreviation list

| **Main Class** | **Sub Class** | **Abbreviation of subclass** |
| --- | --- | --- |
| P-Choline | Lyso‑phosphatidylcholine | LPC |
| P-Choline | Phosphatidylcholine | PC |
| P-Choline | Platelet‑activating factor | PAF |
| P-Ethanol Amine | Lyso‑phosphatidylethanolamine | LPE |
| P-Ethanol Amine | Lysodimethylphosphatidylethanolamine | LdMePE |
| P-Ethanol Amine | Phosphatidylethanolamine | PE |
| P-Ethanol Amine | Dimethylphosphatidylethanolamine | dMePE |
| P-Inositol | Lyso‑phosphatidylinositol | LPI |
| P-Inositol | Phosphatidylinositol phosphates (PI, PIP, PIP₂, PIP₃) | PI / PIP / PIP2 / PIP3 |
| P-Glycerol | Lyso‑phosphatidylglycerol | LPG |
| P-Glycerol | Phosphatidylglycerol | PG |
| P-Serine | Lyso‑phosphatidylserine | LPS |
| P-Serine | Phosphatidylserine | PS |
| P-Acid | Lyso‑phosphatidic acid | LPA |
| P-Acid | Phosphatidic acid | PA |
| P-Acid | Cyclic phosphatidic acid | cPA |
| Cardiolipin | — | CL |
| Glycoglycerollipid | Sufoquinovosyldiacylglycerol | SQDG |
| Glycosphingolipid | Lactotriaosylceramide | GLS |

**References:**

Anders, S., Pyl, P. T., & Huber, W. (2015). HTSeq—a Python framework to work with high-throughput sequencing data. *Bioinformatics, 31*(2), 166-169. doi:10.1093/bioinformatics/btu638

Babraham Bioinformatics. FastQC: a quality control tool for high throughput sequence data, <https://www.bioinformatics.babraham.ac.uk/projects/fastqc/>. Retrieved from <https://www.bioinformatics.babraham.ac.uk/projects/fastqc/>

Dobin, A., Davis, C. A., Schlesinger, F., Drenkow, J., Zaleski, C., Jha, S., . . . Gingeras, T. R. (2013). STAR: ultrafast universal RNA-seq aligner. *Bioinformatics, 29*(1), 15-21. doi:10.1093/bioinformatics/bts635

Faust, J. E., Verma, A., Peng, C., & McNew, J. A. (2012). An inventory of peroxisomal proteins and pathways in Drosophila melanogaster. *Traffic, 13*(10), 1378-1392. doi:10.1111/j.1600-0854.2012.01393.x

Linford, N. J., Bilgir, C., Ro, J., & Pletcher, S. D. (2013). Measurement of Lifespan in Drosophila melanogaster. *Journal of Visualized Experiments*(71), 50068. doi:10.3791/50068
